# Electrotherapy in Pediatric Rehabilitation: A Structured Narrative Review of Clinical Applications, Treatment Parameters, Safety, and Evidence Gaps

**DOI:** 10.3390/jcm15187012

**Published:** 2026-09-10

**Authors:** Andreea Veronica Stavăr, Cristina Octaviana Daia, Brindușa Ilinca Mitoiu, Madalina Codruta Verenca

**Affiliations:** 1 Research Centre in the Medical-Pharmaceutical Field, Medicine and Pharmacy Faculty, “Dunărea de Jos” University of Galati, 800010 Galați, Romania; a.stavar@yahoo.com (A.V.S.); madverrec@gmail.com (M.C.V.); 2 Neuropsychomotor Rehabilitation Department, “St. John” Clinical Emergency Hospital for Children, 800487 Galați, Romania; 3 9th Clinical Department, Medical Rehabilitation, Faculty of Medicine, Carol Davila University of Medicine and Pharmacy, 050474 Bucharest, Romania; 4 Physical Medicine and Rehabilitation Division, Clinical CF2 Hospital, 011464 Bucharest, Romania; 5 Agrippa Ionescu Clinical Emergency Hospital, 011356 Bucharest, Romania

**Keywords:** pediatric rehabilitation, electrotherapy, physical and rehabilitation medicine, transcutaneous electrical nerve stimulation, neuromuscular electrical stimulation, extracorporeal shock wave therapy, repetitive transcranial magnetic stimulation, transcranial direct current stimulation, deep oscillation, laser therapy

## Abstract

**Background/Objectives**: Electrotherapy encompasses diverse therapeutic modalities used in Physical and Rehabilitation Medicine, but pediatric evidence varies substantially across modality–indication pairs. This structured narrative review mapped the available pediatric clinical evidence and provided a clinically structured synthesis of clinical applications, treatment parameters, reported therapeutic outcomes, safety, and evidence gaps in pediatric rehabilitation. **Methods**: PubMed, Scopus, and Web of Science were searched for publications from 1 January 2000 to 17 July 2026. Evidence was synthesized by electrotherapy modality and clinical indication and classified using Oxford Centre for Evidence-Based Medicine (OCEBM) Levels of Evidence. Formal methodological quality and risk-of-bias appraisal using A Measurement Tool to Assess Systematic Reviews 2 (AMSTAR 2) and Risk of Bias 2 (RoB 2) was performed for the evidence underpinning OCEBM Level 1 classifications; no formal GRADE certainty-of-evidence assessment was performed. **Results**: A comparatively more developed pediatric evidence base was identified for radial extracorporeal shock wave therapy (rESWT) in cerebral palsy-related spasticity; transcutaneous electrical nerve stimulation (TENS) and interferential current therapy (IFC) in bladder and bowel dysfunction; and neuromuscular electrical stimulation (NMES)/functional electrical stimulation (FES), repetitive transcranial magnetic stimulation (rTMS), and transcranial direct current stimulation (tDCS) for selected motor rehabilitation outcomes. Photobiomodulation (PBM)/low-level laser therapy (LLLT), pulsed electromagnetic field therapy (PEMF), and high-intensity laser therapy (HILT) showed promising but indication-specific evidence, whereas Transfer of Energy Capacitive and Resistive (TECAR) had preliminary adjunctive evidence. Therapeutic ultrasound and Deep Oscillation Therapy were supported by insufficient pediatric clinical evidence, while several modalities lacked eligible pediatric clinical studies. Short-term tolerability was generally favorable, but long-term safety data remain limited. **Conclusions**: Electrotherapy has an adjunctive role in selected pediatric rehabilitation indications, but prescription should be based on specific modality–indication pairs. The available evidence does not support comparative effectiveness conclusions between modalities. Standardized multicenter trials and long-term safety studies are required.

## 1. Introduction

Pediatric rehabilitation is a rapidly evolving field within Physical and Rehabilitation Medicine (PRM) and is recognized as a distinct area of expertise, with specialized training pathways and established clinical services in countries such as the United States and Canada [1,2].

Across Europe, this discipline continues to expand through specialized rehabilitation programs, multidisciplinary care models, and the growing involvement of PRM specialists dedicated to optimizing function, participation, and quality of life for children with acute and chronic disabling conditions [3,4]. The clinical relevance of pediatric rehabilitation is substantial because childhood neurological, musculoskeletal, congenital, and acquired conditions may result in persistent impairments of mobility, self-care, communication, and participation, with consequences extending into adulthood. Children requiring rehabilitation frequently present with complex and long-term functional needs, emphasizing the importance of effective, safe, and age-appropriate therapeutic interventions.

Conventional rehabilitation approaches, particularly therapeutic exercise, have long formed the foundation of pediatric rehabilitation. In recent years, these interventions have been increasingly integrated with advanced rehabilitation technologies, such as robotics, virtual reality, wearable systems, and digital health solutions, offering promising opportunities to enhance functional recovery and participation in children with disabilities [5,6,7].

Within the medical framework adopted in this review, electrotherapy is regarded as a physician-prescribed therapeutic intervention requiring individualized selection and dosing according to indication, therapeutic target, contraindications, precautions, and modality-specific treatment parameters. When appropriately prescribed and applied, electrotherapy includes non-invasive therapeutic modalities with reported clinical benefits across a broad spectrum of neurological, musculoskeletal, and pain-related conditions in adult rehabilitation [8,9].

Like pharmacological therapy, electrotherapy requires an individualized prescription in which both modality selection and treatment dose are tailored to the patient’s clinical condition and therapeutic objectives. Depending on the specific modality, treatment parameters may include stimulation characteristics, application technique, treatment duration, treatment schedule, and other modality-specific variables that collectively determine therapeutic efficacy and safety [10].

In this review, electrotherapy is broadly conceptualized as the therapeutic use of controlled physical forms of energy, based on modality-specific physiological and pathophysiological mechanisms and delivered according to appropriate treatment parameters and dose. This medical and neurophysiological perspective encompasses both directly applied electrical stimulation and electrically generated mechanical, electromagnetic, or photonic energy. We acknowledge that this broad definition is not universally accepted and that some of these interventions are alternatively classified as electrophysical modalities.

Accordingly, the taxonomy adopted here, including the author-defined category “oscillatory electromechanotherapy,” should be regarded as a clinically and mechanistically oriented framework for this review rather than a universally established classification. Consistent with this expanded conceptual framework, hydrotherapy and natural therapeutic factors were not included, as their primary therapeutic agents do not fall within the controlled physical forms of energy encompassed by the operational definition of electrotherapy adopted in this review. Thermotherapy was not excluded when the thermal effect was generated by an electrically powered therapeutic device; rather, externally applied thermal agents, such as heated water, paraffin, and natural therapeutic factors such as therapeutic mud, were outside the scope of this review. Therapeutic exercise (kinesiotherapy) was likewise considered a distinct rehabilitation intervention based on movement for therapeutic purposes and was not classified as electrotherapy. Although therapeutic exercise and electrotherapy are frequently combined within multimodal rehabilitation programs, their concomitant use does not alter their conceptual distinction. Diagnostic applications of physical energies were also outside the scope of this review, which specifically addresses their therapeutic use in pediatric rehabilitation.

Despite the growing interest in electrotherapy in pediatric rehabilitation, the available evidence remains heterogeneous and unevenly developed across modalities and clinical indications. Existing reviews have predominantly focused on individual electrotherapy modalities, specific pediatric conditions, or selected therapeutic outcomes, rather than providing an integrated appraisal of electrotherapy applications, treatment parameters, safety, and evidence gaps across pediatric rehabilitation. Several studies and evidence syntheses report promising therapeutic effects, but the pediatric literature is frequently characterized by small sample sizes, heterogeneous treatment protocols, variable outcome measures, and insufficiently standardized reporting of treatment parameters and dose. This represents an important clinical gap because electrotherapy should be prescribed as an objective and quantifiable therapeutic intervention, with modality-specific parameters and dose adapted to the therapeutic target and the individual child. Better characterization and standardization of pediatric protocols are therefore essential to both optimize therapeutic efficacy and ensure safety, while potentially expanding evidence-based therapeutic opportunities for children who may benefit from these interventions.

Against this background, this structured narrative review aims to map and critically organize the available pediatric clinical evidence across electrotherapy modalities, including their mechanisms of action, clinical indications, treatment parameters, reported therapeutic outcomes, safety, contraindications, and evidence gaps. Specifically, the review addresses the following questions: Which electrotherapy modalities have been clinically investigated in pediatric rehabilitation and for which indications? What treatment parameters, therapeutic outcomes, and safety considerations have been reported? What is the level of available evidence for each specific modality–indication pair? Which applications remain insufficiently investigated and should be prioritized in future pediatric research? Evidence was therefore assessed at the modality–indication level using the Oxford Centre for Evidence-Based Medicine (OCEBM) Levels of Evidence [11], without undertaking a comparative effectiveness assessment between modalities.

## 2. Materials and Methods

### 2.1. Study Design

This study was conducted as a structured narrative review to map the available pediatric clinical evidence and provide a clinically structured synthesis of the mechanisms of action, clinical indications, treatment parameters, reported therapeutic outcomes, safety considerations, contraindications, and clinical applications of electrotherapy in pediatric rehabilitation. A structured narrative design was selected because the objective was to integrate clinically and mechanistically heterogeneous evidence across multiple electrotherapy modalities, pediatric indications, treatment protocols, outcomes, and safety considerations, rather than to address a single narrowly defined intervention–outcome question or to perform quantitative evidence synthesis. The review was not prospectively designed as a systematic or scoping review; therefore, these methodologies were not retrospectively imposed on the evidence-selection and synthesis process.

Considering the marked heterogeneity of the available literature with respect to study design, investigated pediatric conditions, treatment protocols, and outcome measures, a modality-based descriptive synthesis was adopted. The evidence was analyzed separately for each electrotherapy modality and, within each modality, organized according to the pediatric rehabilitation indications investigated. This approach allowed indication-specific assessment while preserving clinically relevant differences among interventions, populations, treatment protocols, and outcomes.

### 2.2. Literature Search Strategy

A structured literature search was conducted in PubMed, Scopus, and Web of Science for relevant publications published between 1 January 2000 and 17 July 2026, with the final search performed on 17 July 2026. No language restrictions were applied during the literature search or study-selection process. However, all publications ultimately retained for the qualitative synthesis were published in English; therefore, no translation procedure was required.

The search strategy consisted of separate searches combining the full name of each electrotherapy modality with the terms “pediatric rehabilitation” and “children rehabilitation”. The same search approach was applied in PubMed, Scopus, and Web of Science, and modality abbreviations were not used as search terms. The evaluated interventions included therapeutic ultrasound, extracorporeal shock wave therapy (ESWT), transcutaneous electrical nerve stimulation (TENS), interferential current therapy (IFC), neuromuscular electrical stimulation (NMES), threshold electrical stimulation (TES), functional electrical stimulation (FES), repetitive transcranial magnetic stimulation (rTMS), pulsed electromagnetic field therapy (PEMF), photobiomodulation (PBM), low-level laser therapy (LLLT), high-intensity laser therapy (HILT), Multiwave Locked System (MLS) laser therapy, Deep Oscillation Therapy, Super Inductive System (SIS), Transfer of Energy Capacitive and Resistive (TECAR), shortwave diathermy, diadynamic currents (DDCs), and transcranial direct current stimulation (tDCS). The complete database-specific search strings used for each electrotherapy modality in PubMed, Scopus, and Web of Science are provided in Supplementary Table S1 to ensure full reproducibility of the literature search.

Because searches were conducted separately for each electrotherapy modality, database yields were recorded at the modality level. Accordingly, the same publication could be retrieved in more than one database and, in some cases, through more than one modality-specific search. The database yields therefore represent modality-specific search results rather than a single pool of unique records.

Duplicate records retrieved from the three databases were removed before study selection. Following study selection, the retained evidence was analyzed according to each electrotherapy modality and, within each modality, organized according to the pediatric rehabilitation indications investigated, including spasticity, motor impairment, gait dysfunction, upper-limb dysfunction, bladder and bowel dysfunction, pain, musculoskeletal disorders, dysphagia, and other clinically relevant conditions.

The literature search strategy is summarized in Table 1, whereas the database search results and evidence retained for qualitative synthesis are presented in Table 2.

### 2.3. Study Selection

Study selection was independently conducted by two reviewers, with disagreements resolved by consensus. Inter-reviewer agreement statistics and the number of discrepancies were not prospectively recorded; therefore, Cohen’s κ or percentage agreement could not be reliably calculated retrospectively. Titles, abstracts, and full-text publications were assessed according to the predefined eligibility criteria and their relevance to pediatric rehabilitation.

When multiple publications addressed the same modality–indication pair, higher-level evidence was prioritized for establishing the evidence hierarchy, while relevant primary studies were retained when they provided additional or more recent evidence. Newer eligible trials published after the search period of an included systematic review or meta-analysis were considered separately in the qualitative synthesis. Where findings differed between an evidence synthesis and newer primary studies, the discrepancy was interpreted in relation to differences in study populations, intervention protocols, comparators, outcomes, follow-up, and methodological limitations rather than resolved solely according to study-design hierarchy. Full-length conference proceedings were considered only in exceptional circumstances when they provided sufficient methodological and clinical information and represented the only available pediatric evidence for a specific modality or indication, or provided clinically relevant information not reported elsewhere.

### 2.4. Eligibility Criteria

Eligible publications included peer-reviewed full-text articles and evidence syntheses addressing pediatric populations and reporting information on electrotherapy indications, treatment parameters, therapeutic outcomes, safety, tolerability, or contraindications. For the purposes of this review, the pediatric population was defined as individuals younger than 18 years. Primary studies including both pediatric and adult participants were considered only when pediatric data were reported separately. Systematic reviews including children together with young adults were considered when the evidence relevant to the modality–indication pair was predominantly derived from pediatric populations and the pediatric evidence could be clearly identified and interpreted separately. Evidence derived exclusively from adult participants was not used to establish pediatric therapeutic efficacy or assign pediatric OCEBM levels.

Full-length conference proceedings were considered exceptionally when they provided sufficient methodological and clinical information and represented the only available pediatric evidence for a specific modality or indication, or provided clinically relevant information not reported elsewhere. Such publications were used only for descriptive evidence mapping and identification of evidence gaps and were not used independently to establish pediatric therapeutic efficacy or assign an OCEBM Level of Evidence. General mechanistic and safety publications not restricted to pediatric populations were used only to contextualize physical principles, physiological and neurophysiological mechanisms of action, and generally accepted precautions. Such publications were not used to establish pediatric therapeutic efficacy, define pediatric clinical indications, or assign pediatric OCEBM levels.

Editorials, letters, conference abstracts without sufficient methodological or clinical information, duplicate publications, non-peer-reviewed manuscripts, preprints, and publications outside the scope of pediatric rehabilitation were excluded. The reference lists of the included publications were manually screened to identify additional relevant studies.

The methodological quality of this structured narrative review was additionally assessed using the Scale for the Assessment of Narrative Review Articles (SANRA), which evaluates six domains: justification of the article’s importance, statement of concrete aims or formulation of questions, description of the literature search, referencing, scientific reasoning, and appropriate presentation of endpoint data. SANRA was applied as an author self-assessment to improve the methodological transparency and reporting quality of the revised manuscript. The assessment was performed jointly by the two corresponding authors, who reached a consensus SANRA score of 11/12. Item-level scores and brief justifications are provided in Supplementary Table S2. This score represents an author self-assessment and should not be interpreted as an independent methodological validation of the review.

### 2.5. Evidence Assessment

The highest applicable level of evidence was identified separately for each electrotherapy modality–indication pair according to the 2011 OCEBM Levels of Evidence for treatment-benefit questions. Level 1 corresponded to systematic reviews of randomized trials; Level 2 to individual randomized trials; Level 3 to non-randomized controlled cohort/follow-up studies; Level 4 to case series, case–control studies, or historically controlled studies; and Level 5 to mechanism-based reasoning. Classification was performed for each specific modality–indication pair according to the highest applicable level of eligible pediatric clinical evidence. A systematic review was not automatically classified as Level 1 unless it directly addressed the specific modality–indication pair and its underlying evidence met the corresponding OCEBM criteria for treatment-benefit questions. When pediatric clinical evidence was unavailable or insufficient, no OCEBM level was assigned.

OCEBM levels were used descriptively to characterize the hierarchy of available study designs and were not interpreted as measures of evidence certainty, methodological quality, effect magnitude, consistency, or recommendation strength. In interpreting the evidence, particular attention was given to the size and replication of the underlying studies, the predominance of single-center evidence, and potential small-study and publication-bias effects. Findings derived from small or single-center studies were interpreted cautiously and were not considered sufficient, in isolation, to support broad conclusions regarding clinical effectiveness or generalizability. Where systematic reviews or meta-analyses formally assessed publication bias, these findings were incorporated into the qualitative interpretation.

Formal methodological quality and risk-of-bias appraisal of the evidence underpinning OCEBM Level 1 classifications was performed as described in Section 2.7. No formal GRADE certainty-of-evidence assessment was performed.

Given the structured narrative design of this review, no independent calculation or pooling of effect sizes was performed. Where effect estimates, 95% confidence intervals, or minimal clinically important difference thresholds were explicitly reported in the included studies or systematic reviews, these were considered in the qualitative interpretation of clinical relevance. Where such measures were not reported by the original publications, they were not retrospectively derived.

### 2.6. Data Extraction and Evidence Synthesis

Relevant data were extracted regarding study design, pediatric population, clinical indication, electrotherapy modality, treatment parameters, comparator intervention, therapeutic outcomes, adverse events, and principal conclusions.

Study-level characteristics, treatment parameters, treatment schedules, outcomes, follow-up, main findings, and reported adverse events of the included primary pediatric clinical studies are provided in Supplementary Table S3.

Because of the clinical and methodological heterogeneity of the included publications, the evidence was synthesized descriptively by electrotherapy modality. Within each modality, findings were organized according to the pediatric indications investigated, with emphasis on treatment parameters, clinical outcomes, safety, contraindications, and evidence gaps. No quantitative evidence pooling was performed.

### 2.7. Methodological Quality and Risk-of-Bias Assessment of Level 1 Evidence

To provide additional methodological context for the evidence underpinning OCEBM Level 1 classifications, an additional methodological appraisal was performed for all modality–indication pairs assigned OCEBM Level 1. Systematic reviews and meta-analyses directly supporting these classifications were assessed using AMSTAR 2, whereas the relevant randomized controlled trials underpinning the corresponding conclusions were assessed using the Cochrane Risk of Bias 2 (RoB 2) framework. RoB 2 assessments were performed for the outcome relevant to the corresponding modality–indication classification and addressed the effect of assignment to intervention. For crossover randomized trials, the crossover-trial version of RoB 2 was used where applicable. AMSTAR 2 was interpreted according to its domain-based guidance and was not converted into a numerical score.

The appraisal considered methodological features that could materially affect the interpretation of the corresponding Level 1 classification, including protocol registration, comprehensiveness of the literature search, duplicate study selection and data extraction, risk-of-bias assessment, appropriateness of quantitative synthesis, consideration of heterogeneity and publication bias, and methodological limitations of the underlying randomized trials. The resulting judgments were used to inform the methodological interpretation of the evidence underpinning each OCEBM Level 1 modality–indication pair but did not determine or confirm the OCEBM level itself.

This additional appraisal was restricted to the evidence underpinning OCEBM Level 1 classifications and was not intended as a risk-of-bias assessment of the entire evidence corpus. Detailed AMSTAR 2 and RoB 2 judgments are provided in Supplementary Table S4.

**Figure 1:**
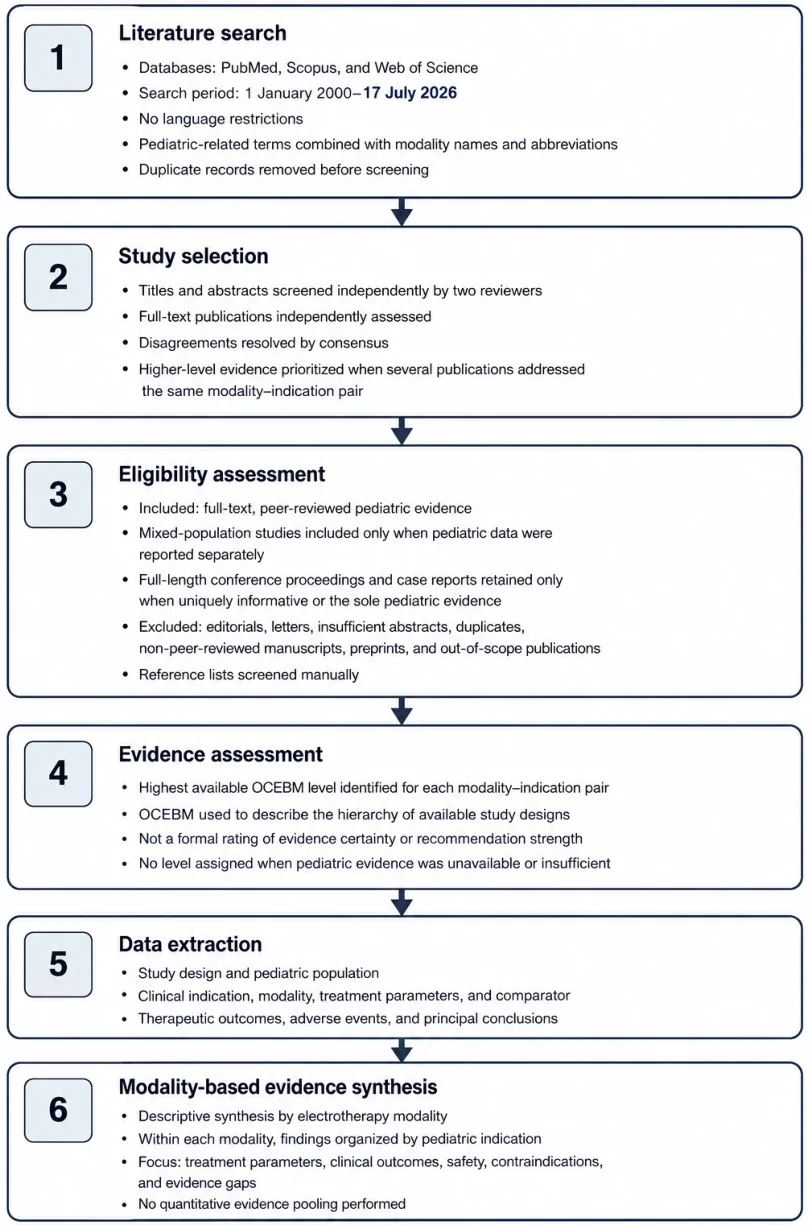
Methodological workflow of the structured narrative review, from literature search and study selection to modality-based evidence synthesis and indication-specific OCEBM assessment.

The review methodology is summarized in Figure 1, illustrating the workflow from the literature search and study selection to modality-based evidence synthesis and indication-specific OCEBM assessment.

## 3. Results

### 3.1. Oscillatory Electromechanotherapy in Pediatric Rehabilitation

The term “oscillatory electromechanotherapy” is used in this review as an author-defined conceptual category for modalities in which electrical energy is converted into therapeutic mechanical waves. Therapeutic ultrasound and extracorporeal shock wave therapy (ESWT) were included in this group because both generate mechanical stimuli through electromechanical transduction and may initiate mechanotransduction pathways [12,13]. Within this framework, intratissular mechanical action is considered the primary therapeutic mechanism. Although these modalities may secondarily generate heat within tissues through the interaction of mechanical energy with biological structures, they are not conceptualized here primarily as thermotherapeutic procedures. This distinction is intended to avoid classifications based predominantly on the resulting endothermic effect and to differentiate these modalities from procedures in which the applied physical energy is used primarily to produce a thermal effect, such as infrared radiation. Accordingly, “oscillatory electromechanotherapy” is used as a pragmatic organizational framework rather than as an established classification of physical agents and does not imply common clinical indications, therapeutic effects, or levels of evidence among the included modalities.

Therapeutic ultrasound must be distinguished from diagnostic ultrasound imaging. Diagnostic ultrasound uses acoustic energy to produce anatomical and functional images, whereas therapeutic ultrasound is intended to induce biological effects in the target tissues [14,15]. No eligible pediatric clinical study provided sufficient evidence of therapeutic effectiveness in rehabilitation. Moreover, experimental evidence has raised concern regarding exposure of active epiphyseal growth plates, although clinically documented growth disturbances in children have not been established [16,17]. Accordingly, therapeutic ultrasound should not be assigned an OCEBM level for a pediatric rehabilitation indication on the basis of the currently available evidence. Studies investigating ultrasound for bone healing represent a distinct therapeutic context and were not used to establish efficacy for conventional therapeutic ultrasound in pediatric rehabilitation. In children, this distinction is particularly important because ultrasound exposure in the vicinity of active growth plates raises specific safety considerations, which should be evaluated separately from evidence concerning ultrasound-assisted bone healing [16].

ESWT includes focused and radial techniques, which differ in wave generation, propagation, penetration depth, and reporting of treatment dose. Radial ESWT (rESWT) predominated in the pediatric rehabilitation literature retained in this review. The proposed rationale in spastic cerebral palsy is principally peripheral: repetitive mechanical stimulation may modify muscle–tendon stiffness and other secondary musculoskeletal alterations associated with chronic spasticity, rather than directly correcting the central neurological lesion [18,19,20,21,22,23]. An early pediatric clinical report published in 2010 described the use of rESWT in children with spastic cerebral palsy [24].

Subsequent randomized trials and evidence syntheses generally reported short-term reductions in clinically assessed spasticity and improvements in passive range of motion after ESWT in children with cerebral palsy [25,26,27,28,29,30,31,32,33,34,35]. Improvements in gait, balance, or gross motor function were also reported in some studies, but these findings were less consistent across outcomes and follow-up periods. The studies were heterogeneous with respect to treated muscles, number of impulses, dose expression, session frequency, concomitant rehabilitation, and outcome assessment. Radial protocols varied substantially in the number of impulses, stimulation frequency, pressure or energy-dose expression, treated muscles, and treatment schedule; study-specific treatment parameters are provided in Supplementary Table S3. Radial ESWT parameters should not be directly extrapolated to focused ESWT, for which dose is generally reported as energy flux density. The available evidence therefore supports an OCEBM Level 1 classification for ESWT in cerebral palsy-related spasticity, while the optimal technique, dose, treatment schedule, and durability of benefit remain uncertain.

Evidence outside cerebral palsy was very limited. A small uncontrolled prospective study that included children with Rett syndrome reported preliminary changes in muscle tone and functional measures after rESWT [36]. Because of the uncontrolled design and small sample, this evidence was classified as OCEBM Level 4 and is insufficient to establish effectiveness.

The current evidence supporting the clinical application of oscillatory electromechanotherapy in pediatric rehabilitation is summarized in Table 3, according to the OCEBM levels of evidence.

### 3.2. Transcutaneous Electrical Nerve Stimulation in Pediatric Rehabilitation

Transcutaneous Electrical Nerve Stimulation (TENS) is a generally well-tolerated and non-invasive electrotherapy modality that modulates peripheral and spinal neural pathways and is widely used for both pain management and neuromodulation of pediatric bladder and bowel dysfunction [37,38,39]. Eleven publications addressing the pediatric clinical use of TENS were retained for the qualitative synthesis [37,38,39,40,41,42,43,44,45,46,47].

The use of TENS to modulate impaired neuromuscular function of the lower urinary tract and bowel is supported by a substantial body of clinical evidence in children with neurogenic bladder (including spina bifida and myelomeningocele), overactive bladder, dysfunctional voiding, bladder and bowel dysfunction, urinary incontinence, constipation, and fecal incontinence [40,41,42,43,44,45].

Across pediatric studies, parasacral TENS delivered over the S2–S3 dermatomes represents the most commonly applied neuromodulation protocol for neurogenic and non-neurogenic bladder and bowel dysfunction. Although stimulation parameters varied among studies, the most frequently used protocol consisted of low-frequency stimulation (10 Hz), pulse durations of approximately 200–300 μs, sensory-level stimulation without visible muscle contraction, and treatment sessions lasting 20–30 min, administered daily or several times per week for 6–12 weeks [37,38,39,40,41,42,43,46,47].

Available pediatric evidence supports parasacral TENS as an adjunctive neuromodulation modality for selected bladder and bowel dysfunctions in children. According to the OCEBM classification, the level of evidence ranges from Level 1 to Level 2 depending on the specific clinical indication, as summarized in Table 4. 

### 3.3. Interferential Current Therapy in Pediatric Rehabilitation

Interferential current therapy (IFC) is a medium-frequency electrotherapy modality in which the interference of two alternating medium-frequency currents generates a therapeutic low-frequency current field within deeper tissues.

Owing to its ability to achieve non-invasive deep neuromodulation with minimal skin discomfort, IFC has been investigated as a therapeutic option for pediatric bladder and bowel dysfunction. Clinical studies have evaluated its use in children with lower urinary tract dysfunction, neurogenic bladder associated with myelomeningocele, non-neuropathic urinary incontinence, bladder and bowel dysfunction, primary nocturnal enuresis, functional constipation, slow-transit constipation, neurogenic bowel dysfunction, and fecal incontinence, while systematic reviews have further supported its safety and potential clinical efficacy [38,48,49,50,51,52].

Treatment parameters varied considerably among studies. Most protocols employed a carrier frequency of approximately 4000 Hz with beat frequencies ranging from 10 to 100 Hz, depending on the therapeutic objective. Electrodes were generally positioned transabdominally or over the pelvic floor and sacral region, with treatment sessions lasting 20–30 min and administered three to five times weekly or as daily home-based therapy. The total treatment duration ranged from 4 weeks to 6 months. However, the marked heterogeneity in stimulation parameters, electrode placement, treatment schedules, and intervention duration currently precludes the establishment of a standardized pediatric treatment protocol for pediatric bladder and bowel dysfunction [38,48,49,50,51,52].

Overall, the available pediatric literature indicates that IFC has been investigated across a broad spectrum of urinary and colorectal disorders, although the strength of evidence differs among clinical indications. The identified indications and their corresponding OCEBM Levels of Evidence are summarized in Table 5.

### 3.4. Neuromuscular Electrical Stimulation and Functional Electrical Stimulation in Pediatric Rehabilitation

Neuromuscular electrical stimulation (NMES) comprises a group of electrotherapeutic techniques designed to activate normally innervated skeletal muscles through electrical stimulation of intact peripheral motor nerves. One of its principal therapeutic applications is Functional Electrical Stimulation (FES), also referred to in part of the rehabilitation literature as Neuromuscular Functional Electrical Stimulation, in which electrically evoked muscle contractions are synchronized with voluntary or task-specific activities to facilitate motor relearning, restore functional movement, and promote neuroplasticity. Contemporary NMES/FES systems are most commonly delivered using biphasic rectangular pulsed currents, which effectively depolarize intact peripheral motor nerves and elicit coordinated muscle contractions [53,54].

In contrast, when the peripheral motor unit is disrupted, as in peripheral nerve injuries or lower motor neuron lesions, conventional NMES/FES is ineffective because the motor nerve is no longer excitable. In these circumstances, muscle contractions must be elicited by direct stimulation of denervated muscle fibers using long-duration, slowly rising waveforms, most commonly exponential currents. This approach, commonly referred to as appropriate electrical stimulation with exponential current, aims to preserve muscle trophicity, limit denervation-induced atrophy, maintain muscle extensibility, and support functional recovery during reinnervation. Understanding the integrity of the peripheral motor unit is therefore essential for selecting the appropriate electrical stimulation modality [53,54].

Historically, one of the earliest applications of NMES in pediatric rehabilitation was Threshold Electrical Stimulation (TES), a low-intensity form of electrical stimulation delivered at or just below the motor threshold, typically during prolonged overnight sessions, with the aim of promoting muscle development and improving motor function in children with cerebral palsy [53,55].

Evidence regarding TES in children with cerebral palsy remains limited. In the available randomized placebo-controlled trial, TES did not demonstrate significant improvements in motor function, muscle growth, spasticity, or other clinically relevant outcomes compared with placebo [55]. Given the limited evidence base and the lack of sufficient confirmatory pediatric studies, no conclusion regarding the clinical effectiveness of TES can currently be established.

Studies of NMES/FES have reported favorable clinical outcomes in both neurological and non-neurological pediatric rehabilitation. In non-neurological conditions, NMES has also been investigated in children with bronchial asthma and type 1 diabetes mellitus. In children with moderate asthma, NMES of the calf muscles added to supervised breathing exercises was associated with improvements in calf muscle strength, exercise tolerance, nocturnal symptoms, and health-related quality of life [56]. In children and adolescents with type 1 diabetes mellitus, a single-group clinical trial investigated the effects of NMES on serum glucose concentration [57]. In a single participant with cerebral palsy and crouch gait, synchronized quadriceps NMES delivered during robotic exoskeleton-assisted walking produced immediate improvements in peak knee extension during stance and total knee excursion in the more affected limb, supporting feasibility rather than establishing clinical efficacy [58]. In children with cerebral palsy or pediatric stroke, NMES/FES has been associated with improvements in gait, lower-limb function, knee stabilization during stance, ankle dorsiflexion, upper-limb motor performance, reaching and grasping ability, and oropharyngeal dysphagia [59,60,61].

Although stimulation protocols varied substantially across the reviewed studies, most contemporary NMES/FES interventions employed biphasic symmetrical pulsed currents with stimulation frequencies of 20–40 Hz, pulse durations of 200–300 μs, and stimulation intensities adjusted to produce a visible functional muscle contraction while maintaining patient comfort. Treatment protocols generally consisted of sessions lasting at least 20 min, performed three to five times per week over intervention periods ranging from 4 to 12 weeks [55,56,57,58,59,60,61].

Overall, the available pediatric evidence supports the use of NMES/FES for selected neurological and non-neurological conditions in which the peripheral motor unit remains intact. The corresponding evidence-based clinical indications and their Oxford Levels of Evidence are summarized in Table 6.

### 3.5. Laser Therapy in Pediatric Rehabilitation

Laser therapy is a non-invasive electrotherapeutic modality that exerts its biological effects through the absorption of light energy by intracellular chromophores, primarily cytochrome c oxidase within the mitochondrial respiratory chain. This process enhances mitochondrial bioenergetics by increasing adenosine triphosphate production and modulating intracellular signaling pathways, thereby enhancing cellular metabolism and reparative capacity. Consequently, laser therapy promotes tissue repair and regeneration while exerting clinically relevant analgesic and anti-inflammatory effects that support functional recovery [62,63].

The laser-based modalities identified in pediatric rehabilitation include photobiomodulation/low-level laser therapy (PBM/LLLT) and high-intensity laser therapy (HILT). Multiwave Locked System (MLS) laser therapy represents a synchronized dual-wavelength photobiomodulation technology; however, pediatric clinical evidence for this specific system has not yet been established.

#### 3.5.1. Photobiomodulation/Low-Level Laser Therapy in Pediatric Rehabilitation

The reviewed literature indicates that photobiomodulation/low-level laser therapy (PBM/LLLT) has been investigated in a limited number of pediatric rehabilitation conditions. In children with spastic cerebral palsy, PBM has shown beneficial effects on spasticity and motor function; however, the evidence remains limited and heterogeneous [64,65]. In polyarticular juvenile idiopathic arthritis, LLLT administered as an adjunct to conventional rehabilitation improved pain, joint stiffness, muscle strength, and functional performance [66]. In children with myelomeningocele, PBM combined with physiotherapy enhanced muscle activation and functional mobility compared with physiotherapy alone [67]. In pediatric Bell’s palsy, LLLT has been associated with accelerated facial nerve functional recovery, although the evidence is currently restricted to a case report [68].

Treatment parameters varied substantially across the included pediatric studies. Most protocols employed red or near-infrared wavelengths ranging from approximately 30 to 500 mW, and energy densities generally ranging from approximately 2 to 9 J/cm^2^ per irradiation point, although higher doses were occasionally used depending on the target tissue and clinical condition. Irradiation was typically applied to multiple anatomical points over the target region, with treatment administered two to three times weekly over periods ranging from 4 to 12 weeks. The considerable heterogeneity in wavelength, power, energy density, irradiation sites, and treatment schedules currently precludes the definition of a standardized PBM/LLLT protocol for pediatric rehabilitation.

The principal clinical applications of PBM/LLLT in pediatric rehabilitation and their corresponding OCEBM Levels of Evidence are summarized in Table 7.

#### 3.5.2. High-Intensity Laser Therapy in Pediatric Rehabilitation

High-Intensity Laser Therapy (HILT) differs from conventional LLLT by delivering substantially higher peak power and greater energy to deeper tissues. The pediatric studies identified in this review employed pulsed neodymium-doped yttrium aluminum garnet (Nd:YAG) laser therapy at a wavelength of 1064 nm, allowing high peak-power delivery while limiting superficial thermal accumulation [69,70].

Pediatric evidence is currently limited to hemophilic arthropathy and juvenile idiopathic arthritis. Three randomized placebo-controlled trials involving 30 [69], 30 [70], and 40 [71] children, respectively (total N = 100), reported that pulsed HILT combined with therapeutic exercise reduced pain and improved selected functional, gait, postural-stability, and plantar weight-bearing outcomes compared with placebo laser plus exercise [69,70,71]. These findings were subsequently supported by a systematic review of randomized rehabilitation trials in children with hemophilia, which identified beneficial effects of laser therapy as an adjunct to conventional physiotherapy on pain and selected functional outcomes [72].

Treatment protocols generally employed pulsed 1064 nm Nd:YAG irradiation three times weekly in combination with therapeutic exercise for 4–12 weeks. Variability in application parameters and total delivered energy currently precludes definition of a standardized pediatric HILT protocol [69,70,71,72].

The available evidence was classified as OCEBM Level 2 for HILT in both pediatric hemophilic arthropathy and juvenile idiopathic arthritis. However, evidence remains condition-specific and based on a limited number of predominantly single-center studies, precluding generalization to other pediatric rehabilitation conditions [69,70,71,72].

The clinical applications of HILT identified in pediatric rehabilitation and their corresponding OCEBM Levels of Evidence are summarized in Table 8.

#### 3.5.3. Multiwave Locked System Laser Therapy in Pediatric Rehabilitation

Multiwave Locked System (MLS) laser therapy combines synchronized continuous 808 nm and pulsed 905 nm infrared emissions, with proposed analgesic, anti-inflammatory, anti-edematous, and tissue-reparative effects. Although MLS has been investigated in adult clinical conditions, treatment protocols remain heterogeneous [73,74].

No eligible peer-reviewed pediatric clinical studies evaluating MLS laser therapy were identified within the predefined search period. Consequently, pediatric clinical indications, treatment parameters, efficacy, and safety cannot currently be established, and no OCEBM level can be assigned.

### 3.6. Electromagnetic Field Therapies in Pediatric Rehabilitation

#### 3.6.1. Repetitive Transcranial Magnetic Stimulation in Pediatric Rehabilitation

Among electrotherapy modalities based on magnetic induction, repetitive Transcranial Magnetic Stimulation (rTMS) generates high-intensity pulsed magnetic fields (Tesla range) that induce electric currents in the cerebral cortex, thereby modulating neuronal excitability and facilitating neuroplasticity. In children, rTMS provides a non-invasive approach for investigating and modulating corticospinal excitability during brain development and has emerged as a promising adjunctive intervention in pediatric neurorehabilitation [75,76,77].

Current evidence indicates that rTMS has been investigated in several pediatric neurological disorders. However, to date, the largest body of rehabilitation evidence has been derived from studies in children with cerebral palsy, where rTMS has been evaluated as an adjunct to conventional rehabilitation for improving motor function, gait, balance, upper limb function, and spasticity. Evidence for other pediatric neurological conditions—including pediatric stroke, autism spectrum disorder, Tourette syndrome, migraine, epilepsy, and attention-deficit/hyperactivity disorder—remains comparatively limited [78,79,80].

The available studies reported short-term improvements in gross and fine motor function, upper-limb performance, manual dexterity, gait, balance, postural control, stereognostic function, and spasticity. Neurophysiological findings further reported modulation of corticospinal excitability, reduced motor thresholds, improved motor evoked potential characteristics, and evidence of enhanced cortical neuroplasticity. These reported improvements were most consistently observed when rTMS was combined with conventional multidisciplinary rehabilitation, particularly constraint-induced movement therapy (CIMT), highlighting its potential role as an adjunctive neurorehabilitation intervention [81,82,83].

Across the reviewed pediatric studies, rTMS was most frequently applied over the primary motor cortex using low-frequency stimulation (predominantly 1 Hz) at intensities corresponding to approximately 80–90% of the resting motor threshold. Most protocols consisted of repeated treatment sessions delivered over several consecutive days or weeks. Available pediatric studies generally reported favorable short-term tolerability, with no serious adverse events reported in the reviewed rehabilitation studies [81,82,83]. However, the limited sample sizes and follow-up durations preclude exclusion of rare adverse events, including seizures, and do not establish long-term safety.

The main clinical indications of repetitive transcranial magnetic stimulation (rTMS) in pediatric rehabilitation, together with the representative pediatric conditions, supporting evidence, and corresponding OCEBM Levels of Evidence, are summarized in Table 9.

#### 3.6.2. Pulsed Electromagnetic Field Therapy in Pediatric Rehabilitation

Pulsed electromagnetic field therapy (PEMF) is a non-invasive electrotherapy modality that delivers low-intensity pulsed electromagnetic fields, typically in the microtesla-to-millitesla range, without direct electrical contact. Depending on the stimulation parameters, PEMF has been associated with analgesic, anti-inflammatory, osteogenic, regenerative, neuromodulatory, and electrobiostimulatory effects, providing the biological rationale for its application in pediatric neurological and musculoskeletal rehabilitation [84,85].

The available pediatric evidence indicates that PEMF has been investigated in a limited number of orthopedic and neurological conditions, including delayed fracture and osteotomy healing, hemophilic arthropathy, and cerebral palsy. Across these indications, PEMF has been associated with improved bone healing, pain reduction, joint function, postural balance, and functional performance, while the available studies consistently reported good tolerability and no serious treatment-related adverse events [85,86,87].

Treatment protocols varied substantially among the reviewed studies. Magnetic field intensities ranged from approximately 150 μT to several millitesla, pulse frequencies from 15 to 200 Hz, and treatment schedules from three sessions weekly for 4–12 weeks to prolonged daily applications. This considerable variability in stimulation parameters currently precludes the establishment of standardized pediatric treatment protocols [85,86,87].

The available pediatric evidence supports PEMF as a promising adjunctive therapeutic modality for selected orthopedic and neurological conditions. However, the limited number of clinical studies precludes standardized evidence-based recommendations for routine pediatric rehabilitation. The corresponding clinical indications and OCEBM Levels of Evidence are summarized in Table 10.

### 3.7. Deep Oscillation in Pediatric Rehabilitation

Deep Oscillation Therapy is a non-invasive electrokinetic modality that uses pulsed electrostatic fields to generate deep tissue oscillations, promoting analgesic, anti-edematous, and tissue-regenerative effects [88].

In pediatric rehabilitation, Deep Oscillation Therapy has been reported in a single case report as part of a multimodal rehabilitation program. Although improvements in edema, pain, and functional recovery were observed, the specific contribution of Deep Oscillation Therapy cannot be isolated from the concomitant interventions; therefore, the current evidence is insufficient to establish its independent efficacy or support routine clinical use [89].

### 3.8. Transcranial Direct Current Stimulation in Pediatric Rehabilitation

Galvanic current is a continuous, unidirectional electrical current used therapeutically through different application methods. In simple galvanization, it is delivered through surface electrodes without an associated therapeutic substance, whereas ionogalvanization (iontophoresis) combines the same current with a therapeutic substance applied at the electrode. These concepts are introduced solely to contextualize the electrotherapeutic basis of tDCS and are not analyzed as separate modalities in this review. From this perspective, tDCS represents a specific transcranial application of low-intensity direct galvanic current, typically 1–2 mA, delivered through scalp electrodes to modulate cortical excitability and facilitate activity-dependent neuroplasticity.

In contrast to rTMS, which induces intracerebral electric currents by rapidly changing magnetic fields, tDCS modifies neuronal membrane polarization without directly eliciting action potentials. Consequently, tDCS is considered an adjunctive neuromodulation technique that enhances the effects of rehabilitation interventions rather than a stand-alone therapeutic modality [90,91,92,93,94].

Current pediatric evidence has focused primarily on children with cerebral palsy, congenital hemiparesis, and perinatal or pediatric stroke. Most clinical studies evaluated tDCS in combination with physiotherapy, occupational therapy, constraint-induced movement therapy (CIMT), robotic rehabilitation, virtual reality, or task-oriented motor training. Across these studies, tDCS was associated with improvements in upper-limb motor function, manual dexterity, gait, balance, postural control, and motor learning. Neurophysiological investigations further reported modulation of corticospinal excitability and enhancement of cortical plasticity, supporting the biological rationale for its use as an adjunctive neurorehabilitation intervention [90,94,95,96,97,98,99].

Beyond motor rehabilitation, tDCS has also been investigated in children with developmental language disorders, persistent cognitive symptoms following concussion, traumatic brain injury, and other neurodevelopmental disorders. Although preliminary studies have reported improvements in language performance and selected cognitive functions, the available evidence remains limited, and larger randomized clinical trials are required before these indications can be routinely recommended in pediatric rehabilitation [100,101,102].

Treatment protocols varied considerably among studies, including anodal, cathodal, and bihemispheric stimulation with different cortical targets and electrode montages. Across the study-specific protocols for which stimulation parameters were available, current intensities ranged from approximately 0.5 to 2 mA, with stimulation typically delivered for approximately 20 min per session; treatment exposure varied from a single experimental session to repeated courses of up to 20 sessions. Across the available pediatric studies, tDCS was associated with a favorable safety profile, with adverse events generally limited to mild transient scalp erythema, tingling, itching, or local discomfort [91,92,93,94,95,96,97,98,99,100,101,102,103]. Current density is an important dosimetric parameter in pediatric tDCS. In the reviewed pediatric protocols for which current intensity and electrode dimensions were available, values were approximately 0.020–0.075 mA/cm^2^ [90]. Importantly, current density should be considered together with age-related anatomical differences when interpreting pediatric tDCS dosing. Therefore, direct extrapolation of adult tDCS dosing to children requires caution [90].

Higher-level evidence is available for selected adjunctive applications of tDCS in pediatric neurological rehabilitation, particularly in cerebral palsy and pediatric stroke. The comparatively more developed evidence concerns upper-limb motor rehabilitation, gait, balance, and motor learning, whereas evidence for cognitive and language rehabilitation remains preliminary. Additional high-quality randomized controlled trials with standardized stimulation protocols are required to establish optimal treatment parameters and long-term clinical efficacy [91,92,94,95,96,97,98,99,100,101,102,103]. The principal pediatric clinical applications of tDCS and the corresponding Oxford Centre for Evidence-Based Medicine (OCEBM) Levels of Evidence are summarized in Table 11.

### 3.9. Transfer of Energy Capacitive and Resistive Therapy in Pediatric Rehabilitation

Transfer of Energy Capacitive and Resistive (TECAR) therapy, also termed capacitive–resistive electric transfer, is a non-invasive radiofrequency diathermy modality typically operating at 0.3–1.2 MHz, although the available operating frequencies vary according to the device. Capacitive and resistive applicators generate different current distributions and may induce thermal and non-thermal effects, including increased tissue temperature and local perfusion [104,105,106,107].

Only one pediatric randomized clinical trial was identified. In 81 children aged 4–16 years with fecal incontinence associated with chronic constipation, TECAR (0.5 MHz, 15 min, twice weekly for six weeks) combined with pelvic-floor biofeedback produced greater improvement in fecal-incontinence severity than biofeedback alone, without additional significant benefits for constipation severity or weekly incontinence frequency [107]. No adverse events were reported in the pediatric trial [107]. Nevertheless, thermal effects require appropriate dose monitoring [106], and interference with cardiac implantable electronic devices has been reported [108].

Accordingly, TECAR combined with pelvic-floor biofeedback may be assigned OCEBM Level 2 evidence for reducing fecal-incontinence severity in children with chronic constipation. Evidence is currently insufficient to establish the efficacy of TECAR monotherapy or its role in other pediatric rehabilitation conditions. The clinical applications of TECAR therapy in pediatric rehabilitation and their corresponding OCEBM Levels of Evidence are summarized in Table 12.

### 3.10. Other Electrotherapy Modalities in Pediatric Rehabilitation

Several electrotherapy modalities routinely used in adult rehabilitation remain insufficiently investigated in pediatric populations. No eligible pediatric clinical studies were identified for Multiwave Locked System (MLS) laser therapy, Super Inductive System (SIS), shortwave diathermy, or diadynamic currents (DDCs) within the predefined search period.

Although these modalities have established physiological and therapeutic rationales in adult rehabilitation, the absence of pediatric clinical studies precludes conclusions regarding their efficacy, safety, treatment parameters, or clinical indications in children. Importantly, the absence of reported pediatric-specific contraindications should not be interpreted as evidence of safety. Further pediatric clinical studies are required before evidence-based recommendations can be established.

### 3.11. Safety, Contraindications, and Precautions of Electrotherapy in Pediatric Rehabilitation

The electrotherapy modalities investigated in children have generally shown favorable short-term tolerability, with predominantly mild and transient adverse effects. However, pediatric safety evidence remains limited by small samples, short follow-up, heterogeneous adverse-event reporting, and exclusion of high-risk patients. Therefore, the absence of reported serious adverse events should not be interpreted as evidence of long-term safety or safety across all pediatric populations [10,77,90,102].

Pre-treatment assessment should consider age and developmental status, ability to communicate discomfort, skin and sensory integrity, relevant comorbidities, and modality-specific risks, including implanted devices, seizure risk, coagulation disorders, photosensitivity, and active growth plates. Treatment parameters should be individualized according to anatomy, tolerance, and device-specific recommendations [10,16,33,77,102,109]. For modalities with limited or absent pediatric evidence, safety cannot be inferred from adult use or from the absence of reported adverse events. The principal reported adverse events, contraindications, precautions, and supporting evidence are summarized in Table 13.

To facilitate clinical interpretation beyond study-design hierarchy alone, the available evidence was additionally summarized using a descriptive evidence-mapping framework that distinguishes relatively developed pediatric evidence, promising but insufficiently replicated applications, investigational applications, and modalities for which pediatric clinical evidence was not identified (Table 14). This classification complements the OCEBM levels and does not represent an assessment of evidence certainty, recommendation strength, or comparative effectiveness.

## 4. Discussion

Within the broad conceptual framework adopted for this review, electrotherapy is conceptualized as the therapeutic use of controlled physical forms of energy, encompassing both directly applied electrical stimulation and electrically generated mechanical, electromagnetic, or photonic energy. Accordingly, this framework comprises conventional electrical stimulation, the author-defined category of oscillatory electromechanotherapy, photobiomodulation, and electromagnetic field-based therapies, while excluding interventions primarily based on therapeutic movement, hydrothermotherapy, and natural therapeutic factors. As acknowledged in the Introduction, this broad taxonomy constitutes a clinically and mechanistically oriented organizational framework developed for the purposes of the present review and should not be interpreted as a universally accepted classification of electrotherapy or electrophysical modalities. Within this framework, electrotherapy is considered a distinct therapeutic domain within PRM, requiring individualized medical prescription based on the clinical indication, therapeutic target, contraindications, precautions, and modality-specific treatment parameters.

The central finding of this review is not that electrotherapy is uniformly effective or ineffective in children, but that the evidence differs substantially across specific modality–indication pairs. Systematic reviews and randomized trials support several applications, whereas others rely on small exploratory studies, isolated reports, or extrapolation from adult practice. Accordingly, OCEBM levels should be interpreted as hierarchies of study design for specific clinical questions rather than measures of evidence certainty, effect magnitude, or recommendation strength. From a clinical perspective, the available evidence should therefore be interpreted according to its relative stage of development rather than as establishing prescribing standards. Some modality–indication pairs have a comparatively developed pediatric evidence base, whereas others remain promising but insufficiently replicated, investigational, or unsupported by pediatric clinical evidence. These categories are intended to guide interpretation of the evidence landscape and should not be regarded as grades of recommendation or treatment endorsement.

An important consideration across the pediatric electrotherapy literature is the predominance of relatively small studies, frequently conducted at single centers. Such evidence may be particularly susceptible to small-study effects, limited external validity, and an increased influence of individual positive studies on the apparent evidence base. In addition, publication bias cannot be excluded, particularly for modality–indication pairs supported by only a few studies, because small studies with neutral or negative findings may be less likely to be published. Accordingly, favorable findings from isolated or small single-center trials were interpreted as preliminary unless supported by independent replication or higher-level evidence.

Among the modalities reviewed, rESWT has one of the comparatively more developed pediatric evidence bases, particularly for cerebral palsy-related spasticity, with systematic reviews and meta-analyses supporting reductions in muscle tone and improvements in range of motion and selected functional outcomes [25,26,27,28,29,30,31,32,33,34,35,36,109]. Its effects are biologically consistent with modulation of the secondary peripheral alterations of chronic spasticity, including fibrosis, increased tissue stiffness, and reduced muscle extensibility [19,23,110]. Nevertheless, protocol heterogeneity limits the definition of an optimal pediatric regimen.

For TENS, particularly parasacral stimulation, relatively more developed pediatric evidence is available for selected bladder and bowel dysfunctions, although outcomes vary across symptoms and populations [39,41,42,43,44,45,46,47]. IFC also shows promising evidence for pediatric pelvic neuromodulation, but heterogeneous protocols and insufficient comparative evidence preclude conclusions regarding superiority over TENS [38,48,49,50,51,52]. NMES/FES has reported favorable effects on gait, upper-limb function, muscle strength, swallowing, and functional performance across selected neurological and non-neurological conditions, contrasting with the limited efficacy of earlier Threshold Electrical Stimulation [53,54,55,56,57,58,59,60,61,62].

PBM/LLLT has shown potential benefits for spasticity and motor function in cerebral palsy, pain and joint function in juvenile idiopathic arthritis, and functional mobility in myelomeningocele, although evidence remains based on relatively few and heterogeneous pediatric studies [63,64,65,66,67,68,69]. Available pediatric evidence suggests potential condition-specific benefits of HILT, particularly for pain and selected functional outcomes in hemophilic arthropathy, while evidence in juvenile idiopathic arthritis remains limited to randomized trial data [70,71,72,73,74].

Non-invasive brain stimulation also shows clinically relevant but outcome-specific evidence. rTMS and tDCS have reported potential benefits in cerebral palsy and pediatric acquired brain injury; however, effects vary according to diagnosis, stimulation parameters, cortical target, concomitant rehabilitation, and outcome domain [77,78,79,80,81,82,83,84,85,90,91,92,93,94,95,96,97,98,99,100,101,102]. Both should therefore be regarded as adjunctive neuromodulation strategies rather than substitutes for active, task-specific rehabilitation. Importantly, statistically significant short-term changes in motor outcome measures should not be equated with clinically meaningful or sustained functional benefit. For both rTMS and tDCS, evidence that reported improvements exceed established clinically meaningful thresholds or persist over longer follow-up periods remains limited, while effects on participation, quality of life, and long-term functional independence are insufficiently characterized.

TECAR has been evaluated in a single pediatric randomized trial as an adjunct to pelvic-floor biofeedback, providing preliminary evidence for reducing fecal-incontinence severity but not establishing efficacy as monotherapy [104,105,106,107,108].

The differences in apparent evidence strength among modalities should be interpreted in the context of both the maturity of the respective research fields and their methodological characteristics. More developed evidence bases generally reflect a larger number of controlled studies, greater replication, and the availability of evidence syntheses, whereas other modality–indication pairs remain supported by isolated or small single-center studies. Apparent positive effects may also be influenced by methodological factors, including small sample sizes, incomplete or difficult blinding inherent to some physical interventions, concomitant rehabilitation therapies, short follow-up periods, and selective reporting of favorable outcomes. These limitations may amplify apparent treatment effects and restrict both causal attribution and generalizability. Consequently, differences in OCEBM level or the number of positive studies should not be interpreted as direct evidence that one electrotherapy modality is intrinsically more effective than another.

From a rehabilitation perspective, the clinical relevance of the reported outcomes should also be interpreted according to their functional level. Changes in spasticity, muscle tone, passive range of motion, muscle strength, or other physiological measures primarily reflect body structure/function outcomes and should not be assumed to translate directly into improved activity or participation. Gait, upper-limb performance, and other task-related outcomes provide evidence at the activity level, whereas participation and quality-of-life outcomes address broader functional impact. Across the reviewed literature, impairment-level and activity outcomes were more frequently investigated than participation or quality-of-life outcomes. Therefore, improvements in physiological or impairment-level measures should be interpreted separately from evidence of functional independence or participation gains.

Important evidence gaps remain. No adequate pediatric clinical evidence was identified for MLS laser therapy, SIS, shortwave diathermy, or DDC, while Deep Oscillation Therapy is supported only by isolated clinical reporting [89]. Although pediatric clinical evidence is currently limited to a single case report, Deep Oscillation Therapy warrants separate consideration as an emerging electrotherapy modality with a distinct therapeutic profile and preliminary favorable tolerability. Its potential role in pediatric rehabilitation requires evaluation in prospective controlled studies with standardized treatment protocols and systematic safety assessment. Therapeutic ultrasound represents a distinct limitation because concerns regarding active epiphyseal growth plates and the absence of adequate pediatric therapeutic trials preclude evidence-based recommendations for routine use [16,17]. These findings indicate absence or insufficiency of evidence rather than evidence of ineffectiveness.

Across modalities, short-term tolerability was generally favorable under the investigated protocols; however, safety reporting and follow-up remain limited, and high-risk children were frequently excluded. The absence of serious reported adverse events therefore cannot establish long-term safety in the developing child [8,9,10,71,90]. Treatment selection should integrate the clinical indication, therapeutic target, developmental characteristics, tissue and peripheral nerve integrity, individual risk factors, and modality-specific precautions.

From a broader rehabilitation perspective, these findings are also consistent with the Learning Rehabilitation System framework, which places individual functioning at the center of rehabilitation and emphasizes person-centred care, environmental context, prevention, and integration of interventions within coordinated rehabilitation pathways [111]. Within this perspective, electrotherapy technologies should not be considered stand-alone interventions, but rather components of individualized, goal-directed rehabilitation programs selected according to the child’s functional needs, clinical context, therapeutic objectives, and available evidence. Their clinical value should therefore be judged not only by changes in impairment-level outcomes, but also by their contribution to meaningful activity, participation, and longer-term functioning.

Future research should prioritize adequately powered multicenter trials, standardized pediatric treatment parameters, systematic adverse-event reporting, longer follow-up, and direct comparisons between modalities used for the same clinical indication. Such studies are required to translate promising modality-specific findings into reproducible, evidence-based electrotherapy prescriptions for pediatric rehabilitation.

### Limitations

This review has several limitations. First, it was designed as a structured narrative rather than a systematic review. Although three major databases were searched using predefined eligibility criteria and two reviewers participated in study selection, evidence prioritization and narrative synthesis involved expert judgment and may have introduced selection and interpretation bias. Although study selection was independently performed by two reviewers and disagreements were resolved by consensus, reviewer-specific screening decisions and discrepancy counts were not prospectively recorded in a form that allowed reliable retrospective calculation of Cohen’s κ or percentage agreement. The protocol was not prospectively registered. In addition, aggregate screening and exclusion counts were not prospectively recorded, precluding reliable retrospective reconstruction of the number of unique records after deduplication, title/abstract exclusions, full-text assessments, and full-text exclusions with reasons. Consequently, a PRISMA-style study-selection flow diagram could not be generated without retrospective reconstruction of unavailable screening data.

Second, the literature search used a relatively focused modality-specific syntax, primarily combining the full name of each electrotherapy modality with “pediatric rehabilitation” or “children rehabilitation,” without systematically incorporating modality abbreviations or broader age-, condition-, and indication-related terminology. Although this approach was consistent with the structured narrative design of the review and was supplemented by reference-list screening, it may have reduced search sensitivity. In particular, some eligible pediatric studies may have been indexed under alternative physical, device-independent, diagnostic, or age-related terminology and therefore may not have been retrieved by the predefined search strings. For example, pediatric HILT studies may be indexed as “pulsed Nd:YAG laser” without using the term “HILT.” Consequently, despite supplementary reference-list screening, incomplete synonym and terminology coverage may have resulted in some relevant pediatric studies being missed and the available evidence for certain modality–indication pairs being underestimated.

Third, formal methodological quality and risk-of-bias appraisal using AMSTAR 2 and RoB 2 was restricted to the systematic reviews and randomized controlled trials underpinning OCEBM Level 1 classifications and was not extended to the entire evidence corpus. No formal GRADE certainty-of-evidence assessment was performed. Effect sizes, confidence intervals, and minimal clinically important difference thresholds were inconsistently reported across the included literature, limiting quantitative comparison of treatment magnitude and clinical relevance across modalities and indications. OCEBM levels were used solely to describe the hierarchy of study designs and should not be interpreted as direct measures of evidence certainty, methodological quality, effect magnitude, consistency, or recommendation strength. Although methodological quality and risk of bias were additionally appraised for Level 1 evidence, this assessment does not eliminate limitations related to inconsistency, imprecision, indirectness, publication bias, or heterogeneity within the underlying evidence. Consequently, a Level 1 classification should not be interpreted as equivalent to high-certainty evidence, evidence of a consistently positive treatment effect, or an automatic recommendation for clinical use.

Finally, substantial heterogeneity existed across populations, diagnoses, treatment parameters, co-interventions, comparators, outcomes, and follow-up periods, precluding quantitative synthesis and meaningful comparative-effectiveness conclusions. Moreover, in a substantial proportion of studies, electrotherapy was delivered in combination with physiotherapy, therapeutic exercise, occupational therapy, CIMT, biofeedback, or other rehabilitation interventions. Unless the study design specifically isolated the added effect of the electrotherapy modality through an appropriate comparator, the observed clinical improvements cannot be attributed to electrotherapy alone. This limits causal attribution and should be considered when translating the reported findings into clinical practice.

The evidence was also concentrated in a limited number of conditions, particularly cerebral palsy and bladder and bowel dysfunction, with many studies being small, single-center, and short-term. This concentration increases the potential influence of small-study effects and limits external validity, while publication bias cannot be excluded, particularly where the evidence for a modality–indication pair is based on only a few studies or a restricted number of research centers. Consequently, favorable findings from small or isolated studies should be interpreted cautiously and require independent replication in adequately powered multicenter trials.

Systematic safety reporting and long-term developmental follow-up were uncommon. Conference proceedings and case reports were retained when they represented the only pediatric evidence; although useful for identifying evidence gaps, these sources provide limited support for efficacy or safety conclusions. Accordingly, the findings should not be generalized beyond the populations, indications, and treatment protocols investigated.

## 5. Conclusions

Electrotherapy has an adjunctive role in selected areas of pediatric rehabilitation, but its use should be based on specific modality–indication pairs rather than on the general concept of “electrotherapy.” Among the modalities reviewed, a comparatively more substantial evidence base—although still heterogeneous and indication-specific—was identified for rESWT in cerebral palsy-related spasticity, parasacral TENS and selected IFC protocols in pediatric bladder and bowel dysfunction, and NMES/FES, rTMS, and tDCS for selected motor rehabilitation outcomes. However, treatment effects and protocols remain heterogeneous, and an OCEBM Level 1 designation should not be interpreted as a universal clinical recommendation.

PBM/LLLT, PEMF, and HILT have promising but indication-specific pediatric evidence, while TECAR has preliminary evidence as an adjunct to pelvic-floor biofeedback. In contrast, evidence for therapeutic ultrasound and Deep Oscillation Therapy remains insufficient, whereas no eligible pediatric clinical evidence was identified for MLS laser therapy, SIS, shortwave diathermy, or diadynamic currents. These evidence gaps preclude recommendations for routine pediatric use.

Electrotherapy should therefore be prescribed as an adjunct within individualized, goal-directed rehabilitation, according to the child’s functional needs, therapeutic target, clinical context, modality-specific risks, and available evidence. Future multicenter trials with standardized pediatric protocols, validated functional outcomes, systematic safety reporting, and long-term follow-up are required to establish broader evidence-based recommendations.

## Figures and Tables

**Table 1:** Overview of the literature search strategy.

Item	Description
Review design	Structured narrative review
Primary databases	PubMed, Scopus, and Web of Science
Search period	1 January 2000–17 July 2026
Language	No language restrictions
Target population	Children and adolescents
Search strategy	Separate modality-specific searches combining the full name of each electrotherapy modality with “pediatric rehabilitation” and “children rehabilitation”
Main search terms	“[Full name of electrotherapy modality] AND pediatric rehabilitation” and “[full name of electrotherapy modality] AND children rehabilitation”
Electrotherapy modalities searched	Therapeutic ultrasound, ESWT, TENS, IFC, NMES, TES, FES, rTMS, PEMF, PBM, LLLT, HILT, MLS laser therapy, Deep Oscillation Therapy, SIS, TECAR, shortwave diathermy, DDC, and tDCS
Evidence synthesis strategy	Modality-based descriptive synthesis with indication-specific organization and OCEBM assessment
Eligible evidence sources	Peer-reviewed full-text articles, systematic reviews, meta-analyses, randomized controlled trials, prospective and observational studies, full-length conference proceedings, and case reports
Additional sources	Manual screening of the reference lists of included publications

Abbreviations: DDC, diadynamic current; ESWT, extracorporeal shock wave therapy; FES, functional electrical stimulation; HILT, high-intensity laser therapy; IFC, interferential current therapy; LLLT, low-level laser therapy; MLS, Multiwave Locked System; NMES, neuromuscular electrical stimulation; OCEBM, Oxford Centre for Evidence-Based Medicine; PBM, photobiomodulation; PEMF, pulsed electromagnetic field therapy; rTMS, repetitive transcranial magnetic stimulation; SIS, Super Inductive System; tDCS, transcranial direct current stimulation; TECAR, Transfer of Energy Capacitive and Resistive; TENS, transcutaneous electrical nerve stimulation; TES, threshold electrical stimulation.

**Table 2:** Modality-specific database search yields and evidence retained for qualitative synthesis.

Electrotherapy Modality	Records Identified in PubMed/Scopus/Web of Science (n)	Evidence Retained for Qualitative Synthesis
Therapeutic ultrasound	131/37/24	2 NR; 2 SR
Extracorporeal shock wave therapy (ESWT)	298/157/101	3 NR; 2 SR; 3 SR/MA; 4 RCTs; 1 PRCT; 1 PCCS; 1 PCS; 1 conference proceeding
Transcutaneous electrical nerve stimulation (TENS)	108/209/102	1 NR; 2 SR; 2 SR/MA; 2 RCTs; 3 PCS; 1 PMS
Interferential current therapy (IFC)	67/83/71	2 RCTs; 3 PCS
Neuromuscular electrical stimulation (NMES)/functional electrical stimulation (FES)	414/224/100	1 SR; 2 RCTs; 2 PCS; 1 FS; 1 SGCT
Photobiomodulation (PBM)/low-level laser therapy (LLLT)	54/42/41	2 NR; 1 SR; 2 RCTs; 1 PRCT; 1 CR
Repetitive transcranial magnetic stimulation (rTMS)	94/32/112	3 NR; 1 SR; 1 SR/MA; 1 RCT; 2 PCS
Pulsed electromagnetic field therapy (PEMF)	14/59/24	1 NR; 1 RCT; 1 PRCT; 1 RCS
Deep Oscillation Therapy	26/3/2	1 CR
High-intensity laser therapy (HILT) *	2/0/2	1 SR; 3 RCTs
Multiwave Locked System (MLS) laser therapy	1/0/0	No eligible pediatric clinical studies identified
Super Inductive System (SIS)	0/0/0	No eligible pediatric clinical studies identified
Transfer of Energy Capacitive and Resistive (TECAR)	1/0/3	1 RCT
Shortwave diathermy	0/0/1	No eligible pediatric therapeutic clinical studies identified
Diadynamic current (DDC)	0/1/0	No eligible pediatric clinical studies identified
Transcranial direct current stimulation (tDCS)	80/281/169	2 SR; 3 SR/MA; 2 RCTs; 1 PRCT; 4 PCS; 1 CR

* HILT-related pediatric studies were additionally identified through terminology-based and reference-list screening, including studies indexed as “pulsed Nd:YAG laser” rather than “HILT”. Abbreviations: CR, case report; DDC, diadynamic current; ESWT, extracorporeal shock wave therapy; FES, functional electrical stimulation; FS, feasibility study; HILT, high-intensity laser therapy; IFC, interferential current therapy; LLLT, low-level laser therapy; MLS, Multiwave Locked System; NMES, neuromuscular electrical stimulation; NR, narrative review; PBM, photobiomodulation; PCCS, prospective case–control study; PCS, prospective clinical study; PEMF, pulsed electromagnetic field therapy; PMS, prospective multicenter study; PRCT, pilot randomized controlled trial; RCS, retrospective case series; RCT, randomized controlled trial; rTMS, repetitive transcranial magnetic stimulation; SGCT, single-group clinical trial; SIS, Super Inductive System; SR, systematic review; SR/MA, systematic review and meta-analysis; tDCS, transcranial direct current stimulation; TECAR, Transfer of Energy Capacitive and Resistive; TENS, transcutaneous electrical nerve stimulation. Note: Database yields represent separate modality-specific searches and should not be interpreted as a single pool of unique records. The same publication could be retrieved in multiple databases and/or through more than one modality-specific search. Duplicate publications were removed during study selection. As aggregate screening counts were not prospectively recorded for this structured narrative review, unique post-deduplication records and stage-specific screening and exclusion counts are not reported.

**Table 3:** Clinical applications of oscillatory electromechanotherapy in pediatric rehabilitation and corresponding Oxford Centre for Evidence-Based Medicine Levels of Evidence.

Oscillatory Electromechanotherapy	Representative Pediatric Pathology	Supporting Evidence	OCEBM Level of Evidence
Therapeutic ultrasound	Pediatric musculoskeletal conditions; insufficient clinical evidence and safety concerns regarding exposure of active epiphyseal growth plates	Insufficient pediatric clinical evidence	Not assignable *
Radial extracorporeal shock wave therapy	Spastic cerebral palsy	SR, MA, RCT	Level 1
Radial extracorporeal shock wave therapy	Rett syndrome	PCS	Level 4

Abbreviations: MA, meta-analysis; OCEBM, Oxford Centre for Evidence-Based Medicine; PCS, prospective clinical study; RCT, randomized controlled trial; SR, systematic review. * Not assignable: Current pediatric clinical evidence is insufficient to assign an OCEBM Level of Evidence because therapeutic ultrasound has not been adequately investigated in children owing to concerns regarding potential effects on open epiphyseal growth plates.

**Table 4:** Clinical indications for transcutaneous electrical nerve stimulation in pediatric rehabilitation and corresponding Oxford Centre for Evidence-Based Medicine Levels of Evidence.

Clinical Indication of TENS	Representative Pediatric Conditions	Supporting Evidence	OCEBM Level of Evidence
Overactive bladder	Urgency, daytime urinary incontinence	SR, MA, RCT, PCS	Level 1
Primary nocturnal enuresis	Monosymptomatic primary nocturnal enuresis	RCT	Level 2
Lower urinary tract dysfunction	Dysfunctional voiding, refractory LUTD	SR, MA, RCT, PCS	Level 1
Neurogenic bladder	Spina bifida, myelomeningocele, neurogenic detrusor overactivity	RCT, PCS, OBS	Level 2
Bladder and bowel dysfunction	Combined urinary and bowel dysfunction	SR, PCS	Level 2
Neurogenic bowel dysfunction	Constipation, fecal incontinence	SR, PCS	Level 2

Abbreviations: LUTD, lower urinary tract dysfunction; MA, meta-analysis; OBS, observational study; OCEBM, Oxford Centre for Evidence-Based Medicine; PCS, prospective clinical study; RCT, randomized controlled trial; SR, systematic review; TENS, transcutaneous electrical nerve stimulation.

**Table 5:** Clinical indications for interferential current therapy in pediatric rehabilitation and corresponding Oxford Centre for Evidence-Based Medicine Levels of Evidence.

Clinical Indication of IFC	Representative Pediatric Conditions	Supporting Evidence	OCEBM Level of Evidence
Lower urinary tract dysfunction	Dysfunctional voiding; refractory lower urinary tract dysfunction	RCT	Level 2
Neurogenic bladder	Neurogenic detrusor overactivity associated with myelomeningocele	RCT	Level 2
Non-neuropathic urinary incontinence	Functional urinary incontinence	RCT	Level 2
Primary nocturnal enuresis	Monosymptomatic primary nocturnal enuresis	RCT	Level 2
Bladder and bowel dysfunction	Combined urinary and bowel dysfunction	PCS, RCT	Level 2
Peripheral circulation enhancement	Hemiplegic cerebral palsy	RCT	Level 2

Abbreviations: IFC, interferential current therapy; OCEBM, Oxford Centre for Evidence-Based Medicine; PCS, prospective clinical study; RCT, randomized controlled trial.

**Table 6:** Clinical indications for neuromuscular electrical stimulation and functional electrical stimulation in pediatric rehabilitation and corresponding Oxford Centre for Evidence-Based Medicine Levels of Evidence.

Clinical Indication of NMES/FES	Representative Pediatric Conditions	Supporting Evidence	OCEBM Levels of Evidence
Gait rehabilitation, lower-limb muscle strengthening and cycling performance, and oropharyngeal dysphagia	Cerebral palsy	SR, RCTs, PS	Level 2
Upper-limb motor impairment (including reaching and grasping dysfunction)	Pediatric stroke, perinatal stroke	PS, CS	Level 3
Serum glucose concentration	Type 1 diabetes mellitus	SGCT	Level 4
Exercise tolerance and health-related quality of life	Bronchial asthma	RCT	Level 2

Abbreviations: CS, case series; FES, functional electrical stimulation; FS, feasibility study; NMES, neuromuscular electrical stimulation; OCEBM, Oxford Centre for Evidence-Based Medicine; PCS, prospective clinical study; PS, prospective study; RCT, randomized controlled trial; RCTs, randomized controlled trials; SR, systematic review.

**Table 7:** Clinical indications for photobiomodulation/low-level laser therapy in pediatric rehabilitation and corresponding Oxford Centre for Evidence-Based Medicine Levels of Evidence.

Clinical Indication of PBM/LLLT	Representative Pediatric Condition	Supporting Evidence	OCEBM Level of Evidence
Spasticity reduction and motor function improvement	Cerebral palsy	SR; PRCT	Level 2
Pain reduction and improvement in joint function and muscle strength	Polyarticular juvenile idiopathic arthritis	RCT	Level 2
Muscle activation and functional mobility improvement	Myelomeningocele	RCT	Level 2
Facial nerve functional recovery	Pediatric Bell’s palsy	CR	Level 4

Abbreviations: CR, case report; LLLT, low-level laser therapy; OCEBM, Oxford Centre for Evidence-Based Medicine; PBM, photobiomodulation; PRCT, pilot randomized controlled trial; RCT, randomized controlled trial; SR, systematic review.

**Table 8:** Clinical indications for high-intensity laser therapy in pediatric rehabilitation and corresponding Oxford Centre for Evidence-Based Medicine Levels of Evidence.

Clinical Indication of HILT	Representative Pediatric Condition	Supporting Evidence	OCEBM Level of Evidence
Pain reduction and improvement in functional capacity, gait, postural control, and weight-bearing	Hemophilic arthropathy	SR; RCTs	Level 2
Pain reduction and functional and gait improvement	Juvenile idiopathic arthritis *	RCT	Level 2

Abbreviations: HILT, high-intensity laser therapy; OCEBM, Oxford Centre for Evidence-Based Medicine; RCT, randomized controlled trial; RCTs, randomized controlled trials; SR, systematic review. * The original HILT publication used the term juvenile rheumatoid arthritis.

**Table 9:** Clinical indications for repetitive transcranial magnetic stimulation in pediatric rehabilitation and corresponding Oxford Centre for Evidence-Based Medicine Levels of Evidence.

Clinical Indication of rTMS	Representative Pediatric Conditions	Supporting Evidence	OCEBM Level of Evidence
Upper-limb motor function improvement	Congenital hemiparesis; unilateral cerebral palsy	SR/MA, RCTs	Level 2
Lower-limb motor function, gait, and balance improvement	Spastic cerebral palsy	SR/MA, RCTs	Level 1

Abbreviations: RCTs, randomized controlled trials; rTMS, repetitive transcranial magnetic stimulation; SR/MA, systematic review and meta-analysis.

**Table 10:** Clinical indications for pulsed electromagnetic field therapy in pediatric rehabilitation and corresponding Oxford Centre for Evidence-Based Medicine levels of evidence.

Clinical Indication of PEMF	Representative Pediatric Conditions	Supporting Evidence	OCEBM Level of Evidence
Bone healing	Delayed fracture union and osteotomy healing	RCS	Level 4
Pain reduction and joint function improvement	Hemophilic knee arthropathy	RCT	Level 2
Postural balance improvement	Spastic cerebral palsy	PRCT	Level 2

Abbreviations: OCEBM, Oxford Centre for Evidence-Based Medicine; PEMF, pulsed electromagnetic field therapy; PRCT, pilot randomized controlled trial; RCS, retrospective case series; RCT, randomized controlled trial.

**Table 11:** Clinical applications of transcranial direct current stimulation in pediatric rehabilitation and corresponding Oxford Centre for Evidence-Based Medicine Levels of Evidence.

Clinical Application	Representative Pediatric Conditions	Supporting Evidence	OCEBM Level of Evidence
Upper-limb motor function and manual dexterity improvement	Unilateral cerebral palsy; congenital hemiparesis; perinatal stroke	SR/MA; RCTs	Level 1
Gait, balance, and motor performance improvement	Spastic cerebral palsy	SR/MA; RCTs; PRCTs	Level 1
Language rehabilitation	Developmental language disorder	PCS	Level 3
Cognitive rehabilitation	Persistent post-concussion symptoms	PCS	Level 3

Abbreviations: MA, meta-analysis; NPS, neurophysiological study; OCEBM, Oxford Centre for Evidence-Based Medicine; PCS, prospective clinical study; PRCT, pilot randomized controlled trial; PS, pilot study; RCT, randomized controlled trial; RCTs, randomized controlled trials; SR, systematic review; SR/MA, systematic review and meta-analysis; tDCS, transcranial direct current stimulation.

**Table 12:** Clinical applications of TECAR therapy in pediatric rehabilitation and corresponding Oxford Centre for Evidence-Based Medicine Levels of Evidence.

Clinical Indication of TECAR	Representative Pediatric Condition	Supporting Evidence	OCEBM Level of Evidence
Reduction in fecal-incontinence severity when combined with pelvic-floor biofeedback	Fecal incontinence associated with chronic constipation	RCT	Level 2
Stand-alone treatment of fecal incontinence or functional constipation	Pediatric fecal incontinence; functional constipation	Insufficient pediatric clinical evidence	Not assignable
Other rehabilitation applications	Pediatric musculoskeletal, neurological, or sports-related conditions	No pediatric clinical evidence	Not assignable

Abbreviations: OCEBM, Oxford Centre for Evidence-Based Medicine; RCT, randomized controlled trial; TECAR, Transfer of Energy Capacitive and Resistive.

**Table 13:** Safety considerations, reported adverse events, contraindications, and precautions of electrotherapy modalities in pediatric rehabilitation.

Electrotherapy Modality	Reported Pediatric Adverse Events	Pediatric-Specific Safety Evidence	General/Device-Related Contraindications or Precautions	Highest Supporting Evidence
Therapeutic ultrasound	No adequate pediatric therapeutic adverse-event data available	Insufficient pediatric clinical safety data	Avoid direct application over active epiphyseal growth plates; consider target anatomy and tissue integrity	Narrative reviews; safety studies
rESWT	Transient pain, local erythema, petechiae/bruising, muscle soreness	Pediatric trials and evidence syntheses indicate generally favorable short-term tolerability under the investigated protocols	Local infection; bleeding/coagulation disorders; suspected malignancy in treatment field; avoid vulnerable neurovascular structures and air-filled organs	SR/MA; RCTs
TENS	Mild tingling, local discomfort, transient skin irritation	Pediatric clinical studies generally report favorable short-term tolerability	Impaired skin integrity or sensation; implanted electronic devices; limited ability to report discomfort	SR/MA; RCTs
IFC	Mild local discomfort, transient skin irritation	Available pediatric clinical studies generally report favorable short-term tolerability	Impaired skin integrity or sensation; implanted electronic devices; limited ability to report discomfort	RCTs; PCS
NMES/FES	Transient skin irritation, muscle soreness, fatigue	Pediatric studies generally report acceptable short-term tolerability; long-term safety data remain limited	Skin and sensory integrity; peripheral motor-unit integrity; joint instability; active thrombosis; implanted electronic devices	SR; RCTs
PBM/LLLT	No consistent serious adverse events reported; occasional transient local reactions	Available pediatric studies indicate favorable short-term tolerability, although safety datasets remain limited	Protective eyewear; avoid direct retinal exposure; photosensitivity and photosensitizing medication	SR; RCTs
HILT	No consistent serious adverse events reported in available pediatric studies	Pediatric trials report favorable short-term tolerability; evidence remains condition-specific and limited	Protective eyewear; avoid direct retinal exposure; photosensitivity; careful dose and tissue-response monitoring	SR; RCTs
rTMS	Headache, scalp discomfort, dizziness, fatigue; rare potential seizure risk	Pediatric studies primarily provide short-term tolerability data; long-term developmental safety has not been established	Seizure-risk and medication screening; intracranial metal; implanted electronic devices; hearing protection	SR/MA; RCTs
tDCS	Tingling, itching, transient scalp erythema, local discomfort, occasional headache	Pediatric studies primarily support short-term tolerability; long-term developmental safety has not been established	Skin lesions at electrode sites; neurological history; skull defects/craniotomy; implanted devices; individualized stimulation parameters	SR/MA; RCTs
PEMF	No consistent serious adverse events reported	Available pediatric studies report favorable short-term tolerability, but the pediatric safety evidence remains limited	Implanted electronic devices; target anatomy; individualized device- and condition-specific assessment	RCTs; pilot RCTs
TECAR	No adverse events reported in the available pediatric RCT	Pediatric safety evidence is currently limited to a single RCT	Thermal tolerance; skin integrity; cardiac implantable electronic devices	RCT
Deep oscillation therapy	No adequate pediatric adverse-event dataset	Pediatric safety evidence is limited to isolated clinical reporting	Device-specific precautions; individualized assessment	CR
MLS laser therapy, SIS, shortwave diathermy, DDC	No pediatric clinical adverse-event data identified	No eligible pediatric clinical safety evidence identified	Modality- and device-specific precautions; pediatric safety should not be inferred from adult use	No eligible pediatric clinical studies identified

Abbreviations: CR, case report; DDC, diadynamic current; FES, functional electrical stimulation; HILT, high-intensity laser therapy; IFC, interferential current therapy; LLLT, low-level laser therapy; MLS, Multiwave Locked System; NMES, neuromuscular electrical stimulation; PBM, photobiomodulation; PCS, prospective clinical study; PEMF, pulsed electromagnetic field therapy; RCT, randomized controlled trial; RCTs, randomized controlled trials; rESWT, radial extracorporeal shock wave therapy; rTMS, repetitive transcranial magnetic stimulation; SIS, Super Inductive System; SR, systematic review; SR/MA, systematic review and meta-analysis; tDCS, transcranial direct current stimulation; TECAR, Transfer of Energy Capacitive and Resistive; TENS, transcutaneous electrical nerve stimulation. Note: Safety considerations and contraindications summarized in this table derive from pediatric clinical evidence where available and from established general safety principles and modality-specific precautions where pediatric evidence is limited. General or adult-derived safety information should not be interpreted as having been independently validated in pediatric populations. Absence of reported serious adverse events should not be interpreted as evidence of established long-term pediatric safety.

**Table 14:** Clinical evidence-mapping classification of electrotherapy applications in pediatric rehabilitation.

Evidence-Mapping Category	Modality–Indication Applications	Clinical Interpretation
Relatively developed pediatric evidence	rESWT—cerebral palsy-related spasticity; TENS—selected bladder and bowel dysfunctions; IFC—selected bladder and bowel dysfunctions; NMES/FES—selected motor rehabilitation applications; rTMS/tDCS—selected cerebral palsy motor outcomes	Multiple pediatric clinical studies and/or higher-level evidence syntheses are available; protocols, populations, outcomes, and follow-up may nevertheless remain heterogeneous.
Promising but insufficiently replicated applications	PBM/LLLT—selected cerebral palsy, juvenile idiopathic arthritis, and myelomeningocele applications; HILT—hemophilic arthropathy and juvenile idiopathic arthritis; PEMF—selected musculoskeletal and neurological applications	Pediatric clinical findings suggest potential benefits, but replication, sample size, protocol standardization, and/or longer-term evidence remain limited.
Investigational applications	TECAR—fecal incontinence as an adjunct to pelvic-floor biofeedback; Deep Oscillation Therapy—selected pediatric rehabilitation application; rESWT—Rett syndrome	Evidence is limited to isolated or very small numbers of pediatric clinical studies and is insufficient to establish routine clinical application.
No identified or insufficient pediatric clinical evidence	Therapeutic ultrasound; MLS laser therapy; SIS; shortwave diathermy; DDC	No eligible pediatric therapeutic clinical evidence was identified, or available evidence was insufficient to support a pediatric clinical application.

Abbreviations: DDC, diadynamic current; FES, functional electrical stimulation; HILT, high-intensity laser therapy; IFC, interferential current therapy; LLLT, low-level laser therapy; MLS, Multiwave Locked System; NMES, neuromuscular electrical stimulation; OCEBM, Oxford Centre for Evidence-Based Medicine; PBM, photobiomodulation; PEMF, pulsed electromagnetic field therapy; rESWT, radial extracorporeal shock wave therapy; rTMS, repetitive transcranial magnetic stimulation; SIS, Super Inductive System; tDCS, transcranial direct current stimulation; TECAR, Transfer of Energy Capacitive and Resistive therapy; TENS, transcutaneous electrical nerve stimulation. Note: This evidence-mapping classification is descriptive and is intended to complement, rather than replace, the OCEBM Levels of Evidence. Categories reflect the relative development and availability of pediatric clinical evidence and should not be interpreted as grades of evidence certainty, recommendation strength, treatment endorsement, prescribing standards, or comparative effectiveness between modalities.

## Data Availability

No new data were created or analyzed in this study. Data sharing is not applicable to this article.

## Supplementary Materials

The following supporting information can be downloaded at: https://www.mdpi.com/article/10.3390/jcm15187012/s1, Table S1. Search strategy and search strings used for each electrotherapy modality; Table S2. SANRA author self-assessment of the structured narrative review; Table S3. Study-level characteristics, treatment protocols, outcomes, and safety of primary pediatric clinical studies; Table S4. Methodological appraisal of OCEBM Level 1 modality–indication pairs using AMSTAR 2 and RoB 2.

## Abbreviations

CIMT	Constraint-induced movement therapy
CP	Cerebral palsy
CR	Case report
DDC	Diadynamic current
ESWT	Extracorporeal shock wave therapy
FES	Functional electrical stimulation
FS	Feasibility study
HILT	High-intensity laser therapy
IFC	Interferential current therapy
LLLT	Low-level laser therapy
LUTD	Lower urinary tract dysfunction
MA	Meta-analysis
MLS	Multiwave Locked System
Nd:YAG	Neodymium-doped yttrium aluminum garnet
NMES	Neuromuscular electrical stimulation
NPS	Neurophysiological study
NR	Narrative review
OBS	Observational study
OCEBM	Oxford Centre for Evidence-Based Medicine
PBM	Photobiomodulation
PCCS	Prospective case–control study
PEMF	Pulsed electromagnetic field therapy
PMS	Prospective multicenter study
PRCT	Pilot randomized controlled trial
PRM	Physical and Rehabilitation Medicine
PS	Prospective study
RCS	Retrospective case series
RCT	Randomized controlled trial
rESWT	Radial extracorporeal shock wave therapy
rTMS	Repetitive transcranial magnetic stimulation
SGCT	Single-group clinical trial
SIS	Super Inductive System
SR	Systematic review
SR/MA	Systematic review and meta-analysis
tDCS	Transcranial direct current stimulation
TECAR	Transfer of Energy Capacitive and Resistive
TENS	Transcutaneous electrical nerve stimulation
TES	Threshold electrical stimulation
