# Supplementary material for: Electrotherapy in Pediatric Rehabilitation: A Structured Narrative Review of Clinical Applications, Treatment Parameters, Safety, and Evidence Gaps
Source: J Clin Med. 2026 Sep 10;15(18):7012. doi: 10.3390/jcm15187012 (PMC13607889; doi:10.3390/jcm15187012)
Supplement: Supplementary file 1 [file jcm-15-07012-s001.zip › jcm-4520773-supplementary.pdf]

Supplementary Table S1. Search strategy and search strings used for each electrotherapy modality.

**Electrotherapy modality**	**Search strings used in PubMed, Scopus, and Web of Science**
Therapeutic ultrasound	therapeutic ultrasound AND pediatric rehabilitation ; therapeutic ultrasound AND children rehabilitation
Extracorporeal shock wave therapy	extracorporeal shock wave therapy AND pediatric rehabilitation ; extracorporeal shock wave therapy AND children rehabilitation
Transcutaneous electrical nerve stimulation	transcutaneous electrical nerve stimulation AND pediatric rehabilitation ; transcutaneous electrical nerve stimulation AND children rehabilitation
Interferential current therapy	interferential current therapy AND pediatric rehabilitation ; interferential current therapy AND children rehabilitation
Neuromuscular electrical stimulation	neuromuscular electrical stimulation AND pediatric rehabilitation ; neuromuscular electrical stimulation AND children rehabilitation
Threshold electrical stimulation	threshold electrical stimulation AND pediatric rehabilitation ; threshold electrical stimulation AND children rehabilitation
Functional electrical stimulation	functional electrical stimulation AND pediatric rehabilitation ; functional electrical stimulation AND children rehabilitation
Repetitive transcranial magnetic stimulation	repetitive transcranial magnetic stimulation AND pediatric rehabilitation ; repetitive transcranial magnetic stimulation AND children rehabilitation
Pulsed electromagnetic field therapy	pulsed electromagnetic field therapy AND pediatric rehabilitation ; pulsed electromagnetic field therapy AND children rehabilitation
Photobiomodulation	photobiomodulation AND pediatric rehabilitation ; photobiomodulation AND children rehabilitation

Low-level laser therapy	low-level laser therapy AND pediatric rehabilitation ; low-level laser therapy AND children rehabilitation
High-intensity laser therapy	high-intensity laser therapy AND pediatric rehabilitation ; high-intensity laser therapy AND children rehabilitation
Multiwave Locked System laser therapy	Multiwave Locked System laser therapy AND pediatric rehabilitation ; Multiwave Locked System laser therapy AND children rehabilitation
Deep Oscillation Therapy	Deep Oscillation Therapy AND pediatric rehabilitation ; Deep Oscillation Therapy AND children rehabilitation
Super Inductive System	Super Inductive System AND pediatric rehabilitation ; Super Inductive System AND children rehabilitation
Transfer of capacitive and resistive energy therapy	Transfer of capacitive and resistive energy therapy AND pediatric rehabilitation ; Transfer of capacitive and resistive energy therapy AND children rehabilitation
Shortwave diathermy	shortwave diathermy AND pediatric rehabilitation ; shortwave diathermy AND children rehabilitation
Diadynamic currents	diadynamic currents AND pediatric rehabilitation ; diadynamic currents AND children rehabilitation
Transcranial direct current stimulation	transcranial direct current stimulation AND pediatric rehabilitation ; transcranial direct current stimulation AND children rehabilitation

Note: Searches were performed using the full name of each electrotherapy modality combined separately with the terms “pediatric rehabilitation” and “children rehabilitation”. Modality abbreviations were not included in the search strings.

Supplementary Table S2. SANRA author self-assessment of the structured narrative review.

SANRA item	Score	Brief justification
**1. Justification of the article's importance**	**2/2**	The Introduction establishes the clinical relevance of electrotherapy in pediatric rehabilitation and identifies the need for an integrated appraisal of clinical applications, treatment parameters, safety, and evidence gaps.
**2. Statement of concrete aims or formulation of questions**	**2/2**	The objective and review questions are explicitly defined and address clinical indications, treatment parameters, safety, and the available evidence.
**3. Description of the literature search**	**2/2**	The Methods describe the databases, search period, search strategy, eligibility criteria, and study-selection process.
**4. Referencing**	**2/2**	Relevant statements and evidence syntheses are supported by appropriate references throughout the review.
**5. Scientific reasoning**	**2/2**	The evidence is critically interpreted according to modality–indication pairs, study design, evidence level, methodological limitations, and safety considerations.
**6. Appropriate presentation of endpoint data**	**1/2**	Endpoint data are reported according to the available studies; however, effect sizes, confidence intervals, and clinically relevant thresholds were not consistently available across the heterogeneous literature.
**Total**	**11/12**	**Joint author self-assessment by the two corresponding authors, reached by consensus.**

Abbreviation: SANRA, Scale for the Assessment of Narrative Review Articles.

Supplementary Table S3. Study-level characteristics, treatment protocols, outcomes, and safety of primary pediatric clinical studies

Modality	Study	Population / sample	Diagnosis / indication	Intervention / comparator	Treatment parameters and protocol	Main outcomes	Follow-up	Main findings	Adverse events
**rESWT**	Onose et al., 2010 [24]	Children with CP; preliminary clinical series	Spastic cerebral palsy	ESWT for spasticity management	NR	Spasticity; clinical motor outcomes	NR	Preliminary pediatric clinical experience suggested reduction of spasticity	NR
**rESWT**	Vidal et al., 2011 [25]	Mixed-age population including pediatric participants	Spasticity	rESWT / placebo	3 treatment sessions at 1-week intervals; rESWT applied to spastic muscle; one treatment arm also received rESWT to the antagonist muscle; exact	Spasticity; passive ROM	NR	rESWT was associated with reduced spasticity and improved passive ROM	No major safety signal reported

					device parameters NR				
**rESWT**	Gonkova et al., 2013 [26]	Children with CP	Muscle spasticity	rESWT / control	1500 shocks; 1.5 bar; 5 Hz; calf muscles; placebo session followed 4 weeks later by active treatment	Muscle tone; ROM	NR	Reduction in muscle spasticity was reported	NR
**rESWT**	Park et al., 2015 [27]	Children with spastic CP	Gastrocnemius spasticity	ESWT; comparison according to treatment-session exposure	1500 shocks; 0.03 mJ/mm²; 4 Hz; medial gastrocnemius; weekly treatment; one group received 1 active + 2 sham sessions, the other 3 active sessions	Spasticity; treatment-duration effects	NR	Treatment effect varied according to treatment-session exposure	NR
**rESWT**	Wang et al., 2016 [28]	Very young children with CP	Spastic plantar-flexor muscles	rESWT / case-control comparison	1500 radial shock waves/session/leg; 0.6 bar; EFD 0.03 mJ/mm²; 8 Hz; gastrocnemius and soleus; 1 session/week for 3 months	Spasticity; plantar-flexor function	NR	Clinical improvement in plantar-flexor spasticity was reported	NR

**rESWT**	Elnaggar, 2019 [29]	Children with spastic CP	Spasticity and motor impairment	ESWT / traditional physical therapy	1200 shocks; 1.5 bar; 5 Hz; calf muscles; ESWT combined with physical training 3 times/week for 3 months	Muscle spasticity; motor function	NR	ESWT group showed improvement in spasticity and motor outcomes	NR
**rESWT**	Bilek & Tekin, 2021 [30]	32 children with unilateral CP	Postural control and balance	ESWT / control	1500 shocks; 1.2 bar; 4 Hz; paravertebral muscles; twice weekly for 8 weeks, combined with neurodevelopmental treatment	Balance; postural control	NR	ESWT was associated with improved balance and postural-control outcomes	NR
**rESWT**	Su et al., 2024 [36]	Patients with spastic CP and Rett syndrome	Spasticity	ESWT	1500 impulses; remaining protocol parameters NR	Clinical spasticity measures; ultrasonographic findings	NR	Clinical and ultrasonographic changes were reported; Rett evidence remained preliminary	NR
**TENS**	Dombek et al.,	Children with myelomeningocele	Neurogenic bladder	TENS	Immediate transcutaneous stimulation; exact	Urodynamic parameters	Immediate	Acute changes in selected	NR

	2019 [40]				electrical parameters NR			urodynamic parameters were evaluated	
**TENS**	Özdemir Ayla et al., 2025 [41]	Children with spina bifida	Neurogenic lower urinary tract dysfunction	Pelvic-floor rehabilitation + TENS / rehabilitation	TENS added to pelvic-floor rehabilitation; 6 weeks; 18 sessions; electrical parameters NR	Urodynamics; clinical urinary outcomes	NR	Addition of TENS was evaluated for urodynamic and clinical effects	NR
**TENS**	Casal-Beloy et al., 2021 [42]	Children with overactive bladder	Overactive bladder	Transcutaneous sacral electrical stimulation / oxybutynin	Sacral transcutaneous electrical stimulation; treatment maintained for 6 months; detailed electrical parameters NR	Urinary symptoms; continence outcomes	NR	Electrical stimulation was compared directly with pharmacological treatment	NR
**TENS**	Maternik et al., 2024 [43]	Pediatric OAB population	Overactive bladder	Parasacral TENS	Repeated parasacral TENS; exact stimulation parameters NR	Immediate and continued urinary outcomes	Continued follow-up	Improvement was reported during and after treatment	NR
**TENS**	Ebert et al., 2022 [46]	Children with OAB	Overactive bladder	Home-based	Repeated home-based parasacral TENS regimen;	OAB symptoms	NR	Practical home-based treatment was	NR

				parasacral TENS	exact parameters NR			associated with clinical improvement	
**TENS**	Coronas Soucheiron et al., 2023 [47]	Pediatric multicenter cohort	OAB refractory to anticholinergic treatment	Sacral TENS	Prospective repeated sacral TENS; exact parameters NR	OAB symptom response	NR	Benefit was reported in selected refractory patients	NR
**IFC**	Kajbafzadeh et al., 2009 [48]	Children with myelomeningocele	Detrusor overactivity and incontinence	Pelvic-floor IFC / comparator	Pelvic-floor interferential stimulation; low-frequency current; pulse duration 250 µs; repetition time 6.6 s; 20 min/session; 18 sessions, 3 times/week	Urodynamic parameters; incontinence	NR	IFC was associated with improvement in selected urodynamic and continence outcomes	NR
**IFC**	Zivkovic et al., 2017 [49]	79 children	Bladder and bowel dysfunction	IFC + diaphragmatic breathing / diaphragmatic breathing / behavioral modification	IFC combined with diaphragmatic breathing; clinic treatment for 2 weeks; breathing exercises and behavioral modification continued at home	Urinary and bowel symptoms	NR	Combined rehabilitation including IFC was associated with improvement in selected outcomes	NR

					for 1 month; exact IFC parameters NR				
**IFC**	Ladi-Seyedian et al., 2019 [50]	46 children	Non-neuropathic urinary incontinence	Pelvic-floor electrical stimulation + muscle training / comparator	Pelvic-floor electrical stimulation combined with muscle training; exact stimulation parameters NR	Urinary continence	NR	Combined electrical stimulation and muscle training improved selected continence outcomes	NR
**IFC**	Abdelhalim & Ibrahim, 2020 [51]	Children with primary nocturnal enuresis	Nocturnal enuresis	Transcutaneous IFC / TENS	Transcutaneous IFC compared with TENS; exact stimulation parameters NR	Nocturnal enuresis frequency; clinical response	NR	Both electrical modalities were evaluated in an RCT	NR
**IFC**	Mohamed et al., 2022 [52]	30 children with hemiplegic CP	Impaired peripheral circulation	Two IFC applications / placebo-control group	Sympathetic or peripheral IFC stimulation; 80–100 Hz; intensity 10–20 mA; 20 min/session; single acute application.	Peripheral circulation	Immediate/temporal	IFC produced measurable temporal effects on peripheral circulation	NR

**TES**	Dali et al., 2002 [55]	Ambulatory children with CP	Motor impairment	Threshold electrical stimulation / placebo	Low-intensity stimulation at or below motor threshold; prolonged overnight stimulation	Motor function; muscle growth; spasticity	NR	No clinically relevant advantage over placebo was demonstrated	NR
**NMES**	Yousef et al., 2022 [56]	Children with bronchial asthma	Reduced exercise tolerance / nocturnal symptoms	Calf-muscle NMES / comparator	Calf-muscle NMES combined with breathing exercise; exact electrical parameters NR	Nocturnal symptoms; exercise tolerance; QoL	NR	Improvement in selected symptoms and quality-of-life outcomes was reported	NR
**NMES**	Fallah et al., 2022 [57]	Children/adolescents with type 1 diabetes	Metabolic response	NMES; single-group study	NMES applied during metabolic/exercise assessment; exact stimulation parameters NR	Serum glucose	NR	Acute metabolic effects were reported	NR
**NMES**	Shideler et al., 2020 [58]	Child with crouch gait due to CP	Knee-extension impairment	NMES integrated with hybrid exoskeleton	NMES to bilateral vastus lateralis and rectus femoris; 25 Hz; pulse width 200 µs; amplitude 36 mA for vastus lateralis and 44	Knee extension; gait biomechanics	NR	NMES improved knee-extension assistance during gait	NR

					mA for rectus femoris; stimulation active during stance and off during swing; approximately 10 min walking per condition				
**FES**	Kapadia et al., 2014 [60]	Children with severe chronic pediatric stroke	Reaching and grasping impairment	FES therapy + rehabilitation	Transcutaneous FES therapy; total treatment exposure approximately 48 h; detailed electrical parameters NR	Reaching; grasping; upper-limb function	NR	Functional upper-limb improvements were reported	NR
**Sensory electrical stimulation**	Umay et al., 2020 [61]	Children with CP and dysphagia	Oropharyngeal dysphagia	Sensory-level electrical stimulation / control	Sensory-level electrical stimulation; exact frequency, pulse width, intensity and schedule NR	Swallowing function	NR	Improvement in selected dysphagia outcomes was reported	NR
**PBM/LLLT**	Zöll et al., 2025 [65]	Children/adolescents with CP	Spasticity	PBM / control; pilot randomized trial	Irradiance 200 mW/cm²; PBM applied over medial and lateral gastrocnemius motor-point	Spasticity; motor function	NR	PBM was associated with changes in spasticity and selected	NR

					regions; preceded by passive gastrocnemius stretching; remaining protocol parameters NR			motor outcomes	
**PBM/LLLT**	Elnaggar et al., 2022 [66]	Children with polyarticular JIA	Pain and joint dysfunction	Low-energy laser + usual treatment / comparator	Low-energy laser therapy applied to the knee joints; wavelength 903 nm; power output 50 mW; energy 1.5 J; combined with the standard exercise program; additional study-specific irradiation/session parameters NR.	Pain; joint function; muscle strength	NR	Improvements in selected pain and functional outcomes were reported	NR
**PBM**	Silva et al., 2023 [67]	Children with myelomeningocele	Reduced muscle activation and mobility	PBM + physiotherapy / physiotherapy	850 nm; continuous wave; 500 mW average radiant power; irradiance 176 mW/cm²; 50 s/site; 9 J/cm²; 25 J/site; 4 sites; 100 J/session; contact technique; 24	Muscle activation; functional mobility	NR	PBM added to physiotherapy was associated with enhanced muscle activation and mobility	NR

					sessions, twice weekly				
**LLLT**	Fontana & Bagnato, 2013 [68]	One 3-year-old child	Bell palsy	LLLT	GaAlAs diode laser; 660 and 780 nm; continuous emission; direct contact; spot area 0.04 cm²; approximately 10 s/point; up to 80 points; 15–30 min/session; 11 sessions over 3 weeks	Facial nerve recovery	Case follow-up	Functional recovery was reported	No major adverse event reported
**HILT**	El-Shamy & Abdelaal, 2018 [69]	Children with hemophilic arthropathy	Pain, gait and functional impairment	Pulsed HILT + exercise / placebo laser + exercise	Pulsed high-intensity Nd:YAG laser; 1064 nm; combined with therapeutic exercise; approximately 3 sessions/week; intervention up to 12 weeks; remaining dose parameters NR	Pain; functional capacity; gait	NR	Addition of HILT improved selected pain, functional and gait outcomes	No consistent serious adverse events reported

**HILT**	El-Shamy et al., 2018 [70]	Children with juvenile rheumatoid/idiopathic arthritis	Pain and functional impairment	Pulsed Nd:YAG + exercise / placebo laser + exercise	1064 nm pulsed Nd:YAG; peak power 3 kW; average power 10.5 W; pulse duration 120–150 µs; frequency 20–30 Hz; energy density 10 J/cm²; treatment area 60 cm²; 15 J/point at 10 points; total energy 1500 J; approximately 8 min/session; 3 sessions/week for 4 weeks	Pain; function; gait	Short-term	Benefits were maintained at short-term follow-up	No consistent serious adverse events reported
**HILT**	Elnaggar, 2020 [71]	Children with hemophilic ankle arthropathy	Pain, postural instability and abnormal weight bearing	Pulsed Nd:YAG + exercise / placebo laser + exercise	1064 nm pulsed Nd:YAG laser; three-phase active treatment; total energy 1500 J; 3 sessions/week for 4 consecutive weeks; combined with a physical exercise program.	Pain; postural stability; plantar weight bearing	NR	Improvement occurred in pain and selected postural/weight-bearing variables; not all balance outcomes differed	No consistent serious adverse events reported

**rTMS**	Gillick et al., 2014 [80]	Children with pediatric hemiparesis	Upper-limb motor impairment	Primed low-frequency rTMS + CIMT / control-CIMT	Primed low-frequency rTMS over the contralesional primary motor cortex; priming at 6 Hz and 90% resting motor threshold for 10 min (600 pulses), immediately followed by 1 Hz rTMS at 90% resting motor threshold for 10 min (600 pulses); 5 rTMS sessions on alternate weekdays over 2 weeks, combined with CIMT.	Upper-limb motor function	NR	Selected adjunctive motor benefits were reported	Short-term tolerability acceptable
**rTMS**	Rich et al., 2016 [81]	Participants from pediatric rTMS + CIMT trial	Stereognosis impairment	Follow-up of rTMS + CIMT trial	Same stimulation protocol as parent Gillick et al. trial	Stereognosis	Follow-up study	Stability of stereognostic outcomes was assessed	NR
**rTMS**	Galabova-Petrova & Ivanov,	Children with hemiplegic CP	Motor dysfunction	Repeated course of low-frequency rTMS +	1 Hz; 90% resting motor threshold; contralesional motor cortex; 1200 pulses/session; 20	Motor function; neuroplasticity	Prospective follow-up	Second course was associated with additional motor/neuro	NR

	2026 [82]			rehabilitation	min/session; 10 consecutive daily sessions excluding weekends per course; two courses separated by 3 months			plastic changes	
**rTMS**	Dadashi et al., 2019 [83]	Children with CP	Balance dysfunction	rTMS + rehabilitation	1 Hz rTMS; 20 min/session; 4 sessions/week for 3 weeks; sham group received the same schedule	Balance control	NR	Changes in balance-control outcomes were reported	NR
**PEMF**	Boyette & Herrera-Soto, 2012 [85]	Children/adolescents	Delayed/nonunited fractures and osteotomies	PEMF	PEMF used for delayed/nonunion fracture or osteotomy; prolonged bone-healing treatment; device-specific frequency/intensity NR	Bone union	Clinical follow-up	Bone-healing outcomes were reported in a retrospective pediatric series	NR
**PEMF**	Eid & Aly, 2015 [86]	Children with hemophilia	Hemarthrosis / hemophilic arthropathy	PEMF / laser therapy	PEMF with the solenoid positioned over both knee joints; 15 Hz; 20 gauss; 20 min/session; 3 sessions/week for	Pain; joint outcomes	NR	Both physical modalities were compared for	NR

					3 consecutive months; combined with conventional physical therapy and compared with LLLT plus the same physical therapy program.			hemarthrosis treatment	
**PEMF**	Pulay et al., 2025 [87]	Children with CP	Dynamic postural-balance impairment	PEMF / sham	Sine waveform; 20/200 Hz; 150 µT; session duration progressively 8, 12 and 16 min; 3 sessions/week for 4 weeks; sham-controlled	Dynamic postural balance	NR	Feasibility established and selected balance changes reported	No consistent serious adverse-event signal
**Deep Oscillation**	Stavar et al., 2025 [89]	Pediatric case	Pediatric disc pathology	Conservative rehabilitation including Deep Oscillation	Deep Oscillation incorporated into multimodal conservative rehabilitation; study-specific frequency/intensity/session parameters NR	Pain; function; clinical evolution	Case follow-up	Clinical improvement reported within multimodal conservative treatment	NR
**tDCS**	Gillick et al.,	20 participants with unilateral CP	Upper-limb motor impairment	Active tDCS + CIMT /	Cathodal tDCS over contralesional	Upper-limb motor function;	NR	Active tDCS was evaluated	Generally favora

	2018 [94]			sham tDCS + CIMT	motor hotspot; anode over contralateral forehead; 0.7 mA; 20 min/session; 10 consecutive weekdays; concurrent CIMT during stimulation plus additional CIMT, total 120 min/day	safety; feasibility		against sham during CIMT	ble short-term tolerability
**tDCS**	Grecco et al., 2017 [95]	Children with ataxic CP	Balance/motor dysfunction	Cerebellar tDCS / sham; crossover pilot	Anodal cerebellar tDCS; 1 mA; electrodes 7 × 5 cm; 20 min/session; 10 sessions; approximately 5 sessions/week; 1-month washout in crossover design	Balance; motor function	NR	Feasibility and motor/balance effects explored	NR
**tDCS**	Hilderley et al., 2025 [96]	Children with hemiparesis/perinatal stroke	Upper-limb impairment	Neuromodulation + motor rehabilitation / comparator	Cathodal tDCS over the contralesional primary motor cortex; two saline-soaked 25-cm² electrodes, with	Upper-limb motor outcomes	NR	Randomized trial evaluated adjunctive neuromodulation	NR

					the cathode over the mapped contralesional motor hotspot and the reference electrode over the contralateral orbit; 1.0 mA with 30-s ramp-up; 20 min/session; daily during 10 days of intensive upper-extremity therapy.				
**tDCS**	Nemanich et al., 2019 [97]	Children with unilateral CP	Corticospinal dysfunction	tDCS + motor training	Same parent intervention: 0.7 mA tDCS; 20 min/session; 10 consecutive weekdays combined with CIMT/motor training	Corticospinal excitability	NR	Neurophysiological modulation following combined treatment was reported	NR
**tDCS**	Metelski et al., 2026 [98]	Children with unilateral spastic CP	Corticospinal excitability	Anodal tDCS	Anodal tDCS over the M1 motor hotspot for the more-affected hand; 2 mA; 20 min; 35-cm² saline-soaked surface electrodes; single session	Recruitment curves; corticospinal responses	NR	Heterogeneous neurophysiological responses observed	NR

					performed between pre- and post-stimulation TMS recruitment-curve assessments; return-electrode location NR.				
**tDCS**	Zhou et al., 2024 [99]	Children with language delay	Language and cognitive impairment	tDCS + language-cognitive training / training comparison	tDCS using 5 × 7 cm electrodes; anode over the left temporal region centered on Wernicke's area and cathode over the right scapula; 1 mA; current density 0.029 mA/cm²; 20 min/session; once daily, 5 sessions/week for 4 weeks; combined with language-cognitive training.	Language; cognition	NR	Combined intervention improved selected language/cognitive outcomes	NR
**tDCS**	de Launay et al., 2022 [100]	12 youth with persistent post-concussion symptoms	Cognitive dysfunction after concussion	tDCS / controlled pilot comparison	Active or sham tDCS paired with dual N-back working-memory task; 3 sessions;	Working memory; cognitive performanc	NR	Feasibility and tolerability established; exploratory cognitive	NR

					exact electrical parameters NR	e; feasibility		effects assessed	
**tDCS**	Ryan et al., 2023 [101]	One adolescent with TBI	Motor-learning impairment	tDCS incorporated into inpatient physiotherapy	tDCS integrated into inpatient physiotherapy program; specific stimulation parameters NR	Motor learning; function	Case follow-up	Feasibility of integrating tDCS into rehabilitation reported	NR
**tDCS**	Fraser et al., 2025 [102]	Pediatric stroke survivors	Tolerability/ safety	tDCS	Bihemispheric tDCS using 5 × 5 cm sponge electrodes; anode over the lesioned motor cortex (C3 or C4) and cathode over the contralesional motor cortex; 20 min/session for 5 consecutive days; day 1 sham, day 2 at 0.5 mA, day 3 at 1.0 mA, days 4–5 at 1.0 mA for participants aged 5–12 years or 1.5 mA for those aged 13–19 years; stimulation delivered during	Tolerability; adverse events	Short-term	Study primarily supported short-term tolerability	No major serious safety signal reported

					occupational therapy.				
**TECAR**	Matlabi Lotfabadi et al., 2025 [107]	81 children, 4–16 years	Fecal incontinence associated with chronic constipation	TECAR + biofeedback / biofeedback alone / standard medical care	0.5 MHz; 15 min/session; twice weekly for 6 weeks	Fecal-incontinence severity; constipation severity; weekly frequency of incontinence	End of 6-week treatment	TECAR + biofeedback produced greater reduction in fecal-incontinence severity than biofeedback alone; no additional significant advantage for constipation severity or weekly incontinence frequency	No adverse events reported

Abbreviations: CIMT, constraint-induced movement therapy; CP, cerebral palsy; EFD, energy flux density; ESWT, extracorporeal shock wave therapy; FES, functional electrical stimulation; GaAlAs, gallium-aluminum-arsenide; HILT, high-intensity laser therapy; IFC, interferential current; JIA, juvenile idiopathic arthritis; LLLT, low-level laser therapy; M1, primary motor cortex; NMES, neuromuscular electrical stimulation; NR, not reported; OAB, overactive bladder; PBM, photobiomodulation; PEMF, pulsed electromagnetic field; QoL, quality of life; rESWT, radial extracorporeal shock wave therapy; rTMS, repetitive transcranial magnetic stimulation; TBI, traumatic brain injury; tDCS, transcranial direct current stimulation; TECAR, transfer of energy capacitive and resistive; TENS, transcutaneous electrical nerve stimulation; TES, threshold electrical stimulation; TMS, transcranial magnetic stimulation.

Supplementary Table S4. Methodological appraisal of OCEBM Level 1 modality–indication pairs using AMSTAR 2 and RoB 2

**Modality–indication**	**Systematic review evidence**	**AMSTAR 2 main considerations**	**RoB 2 findings of underlying randomized evidence**	**Interpretation**	**Final OCEBM**
rESWT – spasticity in cerebral palsy	Kim et al. 2019; Chang et al. 2023	RCT-focused systematic evidence with multi-database searches and quantitative synthesis; limitations in reporting and heterogeneity across protocols	Concerns in some trials regarding allocation concealment, blinding, missing data and selective reporting	Methodological limitations reduce certainty but do not alter the existence of an RCT-based systematic review directly addressing the modality–indication pair	**Level 1 retained**
TENS – urgency/daytime urinary incontinence/OAB	O'Sullivan et al. 2021	SR/MA of 6 randomized controlled studies; relatively small evidence base and heterogeneous protocols	Blinding and allocation procedures variably reported across trials; small samples	Direct RCT-based systematic evidence supports Level 1, with cautious interpretation	**Level 1 retained**
TENS – lower urinary tract dysfunction	O'Sullivan et al. 2021	Same review; directly addresses pediatric LUTS/LUTD	Same limitations; clinical response varied according to comparator and previous treatment	Evidence hierarchy supports Level 1 but does not establish uniform treatment effectiveness	**Level 1 retained**
rTMS – lower-limb/gait/balance in CP	Mansouri et al. 2026	RCT-based systematic review; registered protocol; formal RoB assessment; substantial clinical/protocol heterogeneity	Some concerns particularly regarding randomization/blinding in individual trials	Heterogeneity and absence of pooling for some rTMS outcomes limit certainty	**Level 1 retained**

				but do not remove the systematic RCT evidence	
tDCS – upper-limb/manual dexterity	Fernández-Pérez et al. 2026	PROSPERO-registered RCT-only SR/MA; duplicate screening/extraction; RoB 2; publication-bias and sensitivity analyses	Overall NIBS corpus: 5 low risk, 6 some concerns, 1 high risk	Pooled tDCS effect was not statistically significant and heterogeneity was substantial; Level 1 represents evidence hierarchy, not demonstrated efficacy	**Level 1 retained**
tDCS – gait/balance/motor performance	Saleem et al. 2019; Mansouri et al. 2026	RCT-based systematic/meta-analytic evidence; formal methodological appraisal	Mostly low risk/some concerns depending on trial and outcome	Evidence supports Level 1 hierarchy, but small samples and protocol heterogeneity limit certainty and generalizability	**Level 1 retained**

Abbreviations: AMSTAR 2, A MeaSurement Tool to Assess systematic Reviews 2; CP, cerebral palsy; LUTD, lower urinary tract dysfunction; LUTS, lower urinary tract symptoms; NIBS, non-invasive brain stimulation; OAB, overactive bladder; OCEBM, Oxford Centre for Evidence-Based Medicine; RCT, randomized controlled trial; rESWT, radial extracorporeal shock wave therapy; RoB 2, Risk of Bias 2; rTMS, repetitive transcranial magnetic stimulation; SR/MA, systematic review and meta-analysis; tDCS, transcranial direct current stimulation; TENS, transcutaneous electrical nerve stimulation.
